# Basal forebrain control of wakefulness and cortical rhythms

**DOI:** 10.1038/ncomms9744

**Published:** 2015-11-03

**Authors:** Christelle Anaclet, Nigel P. Pedersen, Loris L. Ferrari, Anne Venner, Caroline E. Bass, Elda Arrigoni, Patrick M. Fuller

**Affiliations:** 1 Division of Sleep Medicine, Department of Neurology, Beth Israel Deaconess Medical Center, Harvard Medical School, Boston, Massachusetts 02215, USA; 2 Comprehensive Epilepsy Clinic, Department of Neurology, Beth Israel Deaconess Medical Center and Harvard Medical School, Boston, Massachusetts 02215, USA; 3 Department of Pharmacology and Toxicology, School of Medicine and Biomedical Sciences, University at Buffalo, Buffalo, New York 14214, USA

## Abstract

Wakefulness, along with fast cortical rhythms and associated cognition, depend on the basal forebrain (BF). BF cholinergic cell loss in dementia and the sedative effect of anti-cholinergic drugs have long implicated these neurons as important for cognition and wakefulness. The BF also contains intermingled inhibitory GABAergic and excitatory glutamatergic cell groups whose exact neurobiological roles are unclear. Here we show that genetically targeted chemogenetic activation of BF cholinergic or glutamatergic neurons in behaving mice produced significant effects on state consolidation and/or the electroencephalogram but had no effect on total wake. Similar activation of BF GABAergic neurons produced sustained wakefulness and high-frequency cortical rhythms, whereas chemogenetic inhibition increased sleep. Our findings reveal a major contribution of BF GABAergic neurons to wakefulness and the fast cortical rhythms associated with cognition. These findings may be clinically applicable to manipulations aimed at increasing forebrain activation in dementia and the minimally conscious state.

## Abstract (web summary)

The mammalian basal forebrain controls cortical rhythm and wake-sleep. Anaclet *et al.* use genetically-targeted chemogenetic systems to activate or inhibit cholinergic, glutamatergic or GABAergic neurons in this region, and reveal their contributions to behavioral and electrocortical arousal in behaving mice.

## Graphical abstract

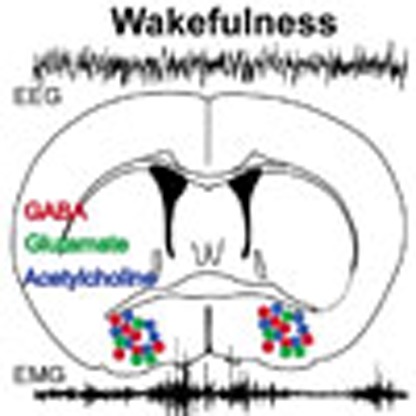

The mammalian basal forebrain (BF) comprises cholinergic and non-cholinergic cell populations that are implicated in a wide range of higher-level neurobiological processes, most fundamentally the support of wakefulness and cortical rhythms associated with cognition^1^,^2^,^3^,^4^,^5^. The specific contribution of individual BF neuronal groups to behavioural and cortical arousal is incompletely understood. Impairment of BF circuitry is associated with cognitive dysfunction in dementia and after head injury, and implicated in the cognitive negative symptoms of schizophrenia^6^,^7^,^8^. The structural and functional integrity of the BF is also necessary for wakefulness and electroencephalographic arousal permitting purposive behaviours and cognition^9^,^10^. Most studies on BF have emphasized cholinergic cortical projections in both normal physiology^11^ and disease states^12^. Cholinergic cell loss in Alzheimer's disease and dementia with Lewy bodies, along with cortical slowing and cognitive impairment that result from centrally acting anti-cholinergic drugs, has reinforced this hypothesis. Paradoxically, experimental lesions of cholinergic BF neurons produce limited changes in arousal, including sleep–wake cycles^13^,^14^, whereas lesions of all neuronal groups together result in stupor or coma^9^, suggesting a potentially crucial role for either GABAergic or glutamatergic BF neurons in these processes.

Using genetically-targeted chemogenetic systems, we sought to re-examine the specific contribution of coextensive cholinergic, glutamatergic and GABAergic BF cells groups^15^,^16^ in supporting wakefulness and fast cortical rhythms in behaving mice. The findings reported here demonstrate a previously unidentified contribution of BF GABAergic neurons to wakefulness and high-frequency electroencephalographic (EEG) activity associated with cognition. In contrast, intermingled cholinergic and glutamatergic neurons of the BF appear to play a lesser role in promoting wakefulness, but do help suppress cortical slowing.

## Results

### AAV-mediated expression of chemogenetic systems in mouse BF

**Figure 1:**
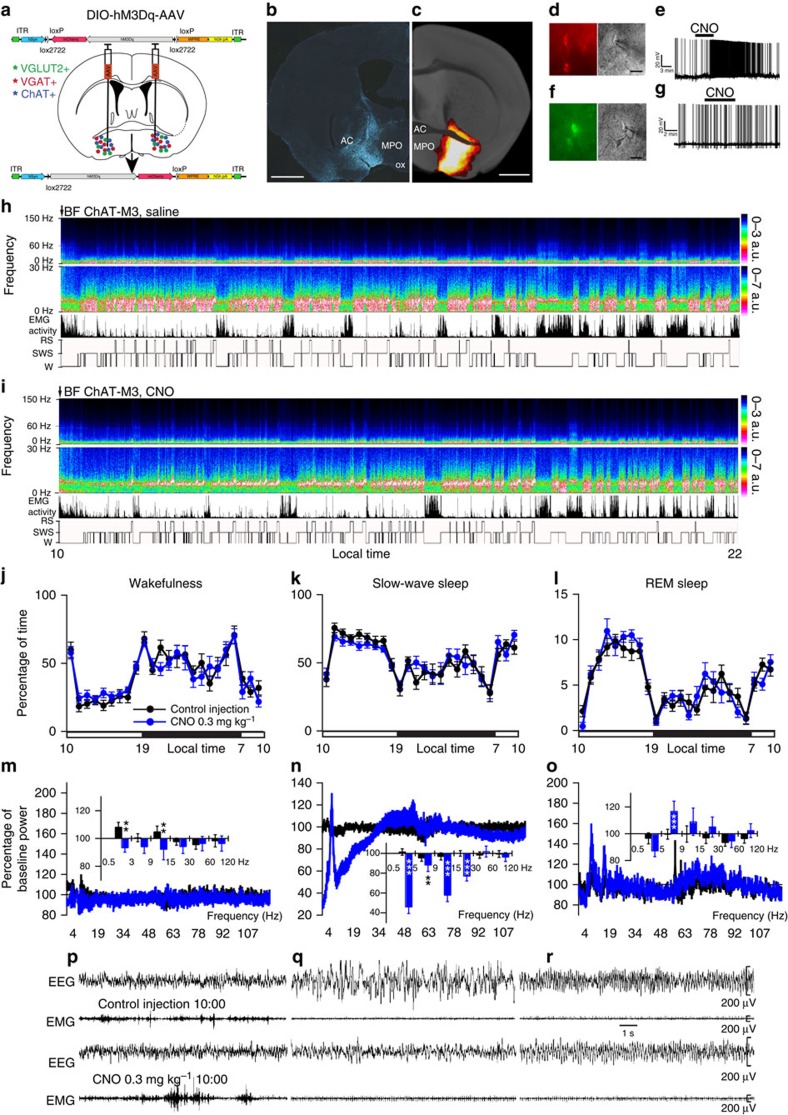
Administration of CNO activates cholinergic BF hM3Dq^+^ neurons and produced a generalized decrease in the slow-wave sleep (SWS) EEG power density. (**a**) Experimental design: bilateral injections of DIO-hM3Dq-AAV were placed into the BF of *ChAT-IRES-Cre*, *Vglut2-IRES-Cre* or *Vgat-IRES-Cre* mice, resulting in the expression of hM3Dq in BF cholinergic, glutamatergic or GABAergic neurons, respectively. (**b**) Coronal section from injected *ChAT-IRES-Cre* mouse showing hM3Dq-expressing BF cholinergic (mCherry^+^) neurons (scale bar, 1 mm). (**c**) Heat map generated from ten injection cases in the *ChAT-IRES-Cre* mouse; white colour=area of maximum overlap of hM3Dq-AAV transduction/expression across ten injection cases. (**d**) hM3Dq-expressing BF cholinergic neurons (left) visualized under IR-DIC (right) during whole-cell recordings (scale bar, 20 μm) showed a strong depolarizing and firing response to bath application of CNO (500 nM (**e**)). (**f**) Non-hM3Dq-expressing cholinergic BF neurons, recorded from *ChAT-IRES-cre* mice (scale bar, 20 μm) with *hr*GFP-AAV control injections did not respond to CNO (**g**). (**h**,**i**) Example compressed spectral array (CSA; 0–30 Hz and 0–150 Hz), EMG activity and hypnogram over 12 h following vehicle (**h**) or CNO (0.3 mg kg^−1^, IP; ZT3 (**i**)) administration in a mouse with bilateral hM3Dq receptor expression in BF cholinergic neurons. The power of the slow EEG frequencies, in particular *δ* and *θ* (0.5–9 Hz), was markedly and uniquely decreased as compared with vehicle administration in the same mouse. (**j**–**l**) Hourly sleep–wake amounts (±s.e.m.) following injection of CNO (0.3 mg kg^−1^, IP, ZT3=10 A.M., *n*=13 mice) or vehicle. (**m**–**o**) Power spectrum changes (±s.e.m.) over baseline during the 3-h post-injection period for vehicle injection as compared with the 3-h post-injection period for CNO (0.3 mg kg^−1^, IP, ZT3=10 A.M., *n*=9 mice) and the quantitative changes (±s.e.m.) in power for the *δ* (0.5–3 Hz for W and 0.5–5 Hz for SWS and REM sleep), *θ* (3–9 or 5–9 Hz), *α* (9–15 Hz), *β* (15–30 Hz), low *γ* (30–60 Hz) and high *γ* (60–120 Hz) frequency bands following vehicle or CNO administrations. (**p**–**r**) EEG/EMG examples of wake (W), SWS and REM sleep (RS) from the first hour post injection of saline (top) or CNO (bottom) in a mouse with bilateral hM3Dq receptor expression in BF cholinergic neurons. The raw EEG and EMG traces following CNO injection in *q* provide unambiguous evidence of the existence of SWS when BF cholinergic neurons are activated. Note, however, that as compared with vehicle injection, SWS after CNO injection is characterized by decreased slow-wave activity (delta band, *n*) density. AC=anterior commissure; MPO=medial preoptic area; ox=optic chiasm. A two-way analysis of variance using the between-subjects factor of injection (vehicle or CNO) and the within-subjects factors of time of day or frequency band was used to analyse the percentage(s) of time spent in W, SWS and REM sleep or the frequency bands during W and SWS; *P<0.05, **P<0.01 and ***P<0.001; *P*≥0.05=not significant (NS).

We achieved acute and selective activation of BF cholinergic (ChAT), glutamatergic (VGLUT2^+^) or GABAergic (VGAT^+^) cell groups in behaving mice by placing bilateral injections of a Cre-recombinase-enabled chemogenetic activating system (hSyn-DIO-hM3Dq-mCherry; Fig. 1a) into the BF of *ChAT-IRES-Cre*, *Vglut2-IRES-Cre* or *Vgat-IRES-Cre* mice, respectively, using an adeno-associated viral (AAV) vector delivery system (hM3Dq-AAV). Within the BF, and for all three cell populations targeted, hM3Dq-expressing somata were observed in the substantia innominata, horizontal limb of the diagonal band, magnocellular preoptic nucleus and ventral pallidum and, in some *ChAT-IRES-Cre* and *Vgat-IRES-Cre* mice, variably within the inferior medial part of the globus pallidus (GP) and ventral medial and posterior lateral divisions of the bed nucleus of the stria terminalis (Supplementary Fig. 1). By contrast, no hM3Dq-expressing neurons were found in the medial septum, vertical limb of the diagonal band or the neighbouring lateral or medial preoptic areas of any experimental mice. Physiologically, in the absence of the selective hM3Dq ligand, clozapine-N-oxide (CNO) there were no differences in hourly sleep–wake or power distribution in baseline behavioural sleep between *ChAT-IRES-Cre*, *Vglut2-IRES-Cre* or *Vgat-IRES-Cre* mice expressing hM3Dq in BF neurons and non-hM3Dq-expressing littermate mice (Supplementary Fig. 2 and Supplementary Table 1). Moreover, injections of CNO at a dose (0.3 mg kg^−1^, intraperitoneal (IP)) used previously^17^ were without significant effect on these parameters in non-hM3Dq-expressing mice (Supplementary Fig. 3 and Supplementary Table 2).

### Activation of cholinergic BF neurons

The BF cholinergic population was studied first. Robust cell-surface expression of hM3Dq receptors was observed in the BF of *ChAT-IRES-Cre* mice (Fig. 1b,d) and was absent in non-cre-expressing littermates (Supplementary Table 3), confirming the absolute requirement for cre activity to enable hM3Dq expression on BF cholinergic somata. Whole-cell current clamp recordings of BF cholinergic neurons expressing hM3Dq showed the expected depolarization (+6.4±1.2 mV; *n*=7; *P*<0.01 paired *t*-test control versus CNO) and increased firing response to CNO (500 nM), when applied *in vitro*. These effects were reversible following CNO washout (Fig. 1d,e) and CNO application had no effect on non-hM3Dq-expressing BF cholinergic neurons (resting membrane potential in control −46.0±2.6; in CNO: −46.7±1.9 mV; *n*=6; *P*=0.27 paired *t*-test), confirming that CNO-mediated activation of BF cholinergic neurons occurs only in neurons expressing hM3Dq receptors (Fig. 1f,g). Strong activation was also indicated by robust c-Fos expression in hM3Dq^+^ cells following CNO administration (Supplementary Table 3). We next studied the effect of BF cholinergic cellular activation in behaving mice. Following vehicle injections at 1000 hours (Zeitgeber time: ZT3, 3 h after lights on), these mice showed a typical hypnogram with long bouts of slow wave sleep (SWS) during the light period, marked by low electromyogram (EMG) activity and high EEG slow-wave activity (Fig. 1h). Following CNO administration, a significant increase in both wake and SWS bout number during the entire light period (Supplementary Table 4) was observed, indicating sleep fragmentation. The onset of rapid eye movement (REM) sleep was delayed, yet total time spent in REM sleep was unaltered (Fig. 1l and Supplementary Table 4). CNO-induced activation of BF cholinergic neurons also depressed lower frequency EEG activity (below 30 Hz) during SWS (Fig. 1i,n), an effect so pronounced that it was evident in the raw EEG (Fig. 1q). The same results were obtained when CNO injections were performed at the beginning of the dark period, a time of maximal wakefulness in mice (Supplementary Fig. 4a,c,e,g,i and Supplementary Table 5), further demonstrating that activation of BF cholinergic neurons has a destabilizing effect on SWS.

### Activation of glutamatergic BF neurons

**Figure 2:**
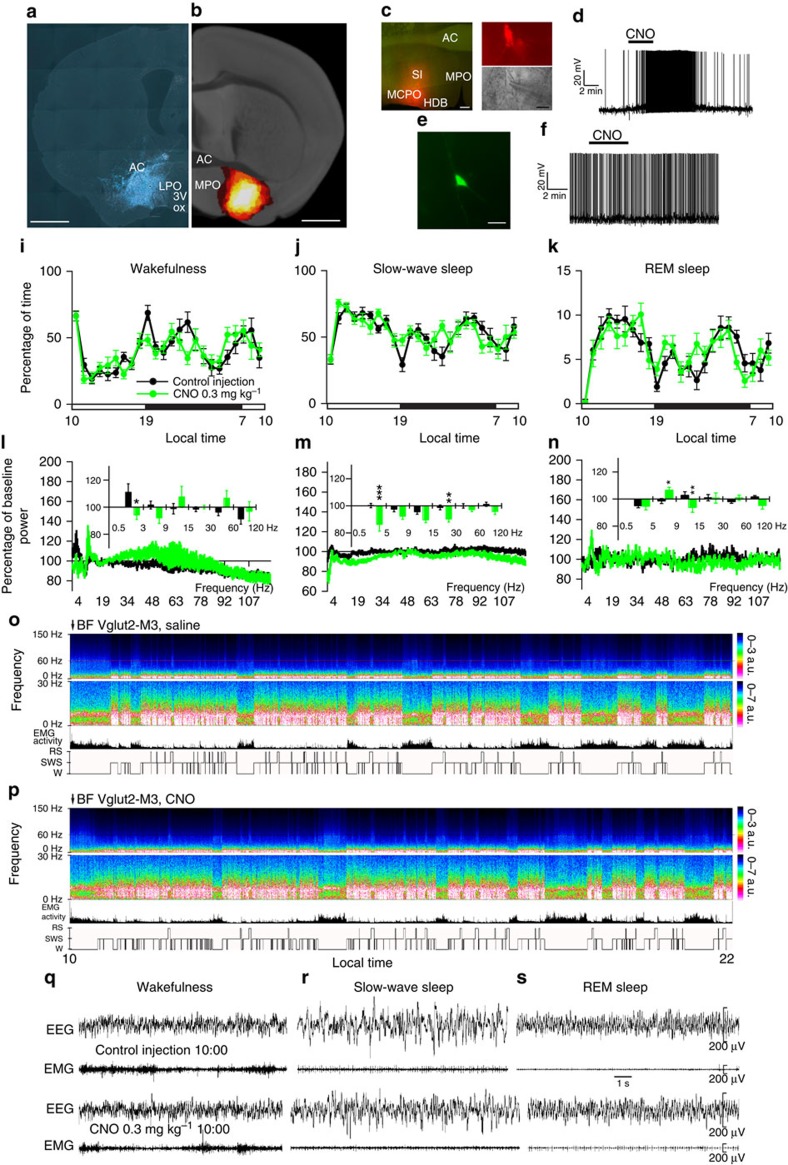
Administration of CNO activates glutamatergic BF hM3Dq^+^ neurons, but had little effect on sleep–wake or EEG power density. (**a**) Coronal section from injected *Vglut2-IRES-Cre* mouse showing hM3Dq-expressing BF glutamatergic (mCherry^+^) neurons (scale bar, 1 mm). (**b**) Heat map generated from ten injection cases in the *Vglut2-IRES-Cre* mouse; white colour=area of maximum overlap of hM3Dq-AAV transduction/expression across ten injection cases. (**c**) Region of BF containing the recorded hM3Dq-expressing glutamatergic neurons (left panel, native mCherry-hM3Dq signal, scale bar, 300 μm) and a representative hM3Dq-expressing BF glutamatergic neuron (top right panel) visualized under IR-DIC (bottom right panel) during whole-cell recordings (scale bar, 10 μm). (**d**) Whole-cell current clamp recordings of BF glutamatergic neurons expressing hM3Dq showed the expected depolarization and increased firing response to CNO (500 nM) when applied *in vitro*. (**e**,**f**) Non-hM3Dq-expressing glutamatergic BF neurons, recorded from *Vglut2-IRES-Cre* mice injected with *hr*GFP-AAV (scale bar, 30 μm) did not respond to CNO. (**i**–**k**) Hourly sleep–wake amounts (±s.e.m.) following injection of CNO (0.3 mg kg^−1^, IP, ZT3=10 A.M., *n*=11 mice) or vehicle. (**l**–**n**) Power spectrum changes (±s.e.m.) over baseline during the 3-h post-injection period for vehicle injection as compared with the 3-h post-injection period for CNO (0.3 mg kg^−1^, IP, ZT3=10 A.M., *n*=9 mice) and the quantitative changes (±s.e.m.) in power for the *δ* (0.5–3 Hz for W and 0.5–5 Hz for slow-wave sleep (SWS) and REM sleep), *θ* (3–9 or 5–9 Hz), *α* (9–15 Hz), *β* (15–30 Hz), low *γ* (30–60 Hz) and high *γ* (60–120 Hz) frequency bands following vehicle or CNO administrations. (**o**,**p**) Example CSA (0–30 Hz and 0–150 Hz), EMG activity and hypnogram over 12 h following vehicle (**o**) or CNO (0.3 mg kg^−1^, IP; ZT3; (**p**)) administration in a mouse with bilateral hM3Dq receptor expression in BF glutamatergic neurons. A black bar (4–6 bins=1–1.5 Hz large) was inserted to mask the 60 Hz contamination of the EEG recordings. (**q**–**s**) EEG/EMG examples of wake (W), SWS and REM sleep (RS) from the first hour post injection of saline (top) or CNO (bottom) in a mouse with bilateral hM3Dq receptor expression in BF glutamatergic neurons. The raw EEG and EMG traces following CNO injection provide unambiguous evidence of the existence of the three sleep–wake stages while BF glutamatergic neurons are activated. AC, anterior commissure; CPu, caudate putamen; HDB, diagonal band of Broca; MCPO, magnocellular pre-optic nucleus; MPO, medial preoptic area; SI, substantia innominata; ox, optic chiasm. A two-way analysis of variance using the between-subjects factor of injection (vehicle or CNO) and the within-subjects factors of time of day or frequency band was used to analyse the percentage(s) of time spent in W, SWS and REM sleep or the frequency bands during W and SWS; *P<0.05, **P<0.01 and ***P<0.001; *P*≥0.05=not significant (NS).

We next sought a similar characterization of the BF glutamatergic cell population. Robust cell-surface expression of hM3Dq receptors was observed in the BF of *Vglut2-IRES-Cre* mice (Fig. 2a,c,d) and was absent in non-cre-expressing littermates. As with the cholinergic cell group, we observed the expected depolarization (+6.5±1.4 mV; *n*=6; *P*<0.01 paired *t*-test control versus CNO) and increased firing response to CNO (500 nM) when applied *in vitro*, as well as reversal upon washout (Fig. 2c,d), with CNO application having no effect on non-hM3Dq-expressing BF glutamatergic neurons (47.3±3.5 mV versus −47.2±3.5 mV; *n*=7; *P*=0.89 paired *t*-test; Fig. 2e,f). Injections of CNO also potently drove c-Fos expression in hM3Dq^+^ neurons in the BF (Supplementary Table 3). In behaving mice, CNO-induced activation of BF glutamatergic neurons produced a significant effect in the cortical EEG during SWS (decrease in both *δ* and *β* power bands (Fig. 2m,p), but were without effect on sleep–wake quantity, consolidation and sleep latency (Fig. 2i,l and Supplementary Table 4), although a non-significant trend towards an increase in sleep latency was noted. These results indicate that activation of BF glutamatergic neurons also has a destabilizing effect on sleep, albeit to a lesser degree than that observed following BF cholinergic activation.

### Activation of GABAergic BF neurons

**Figure 3:**
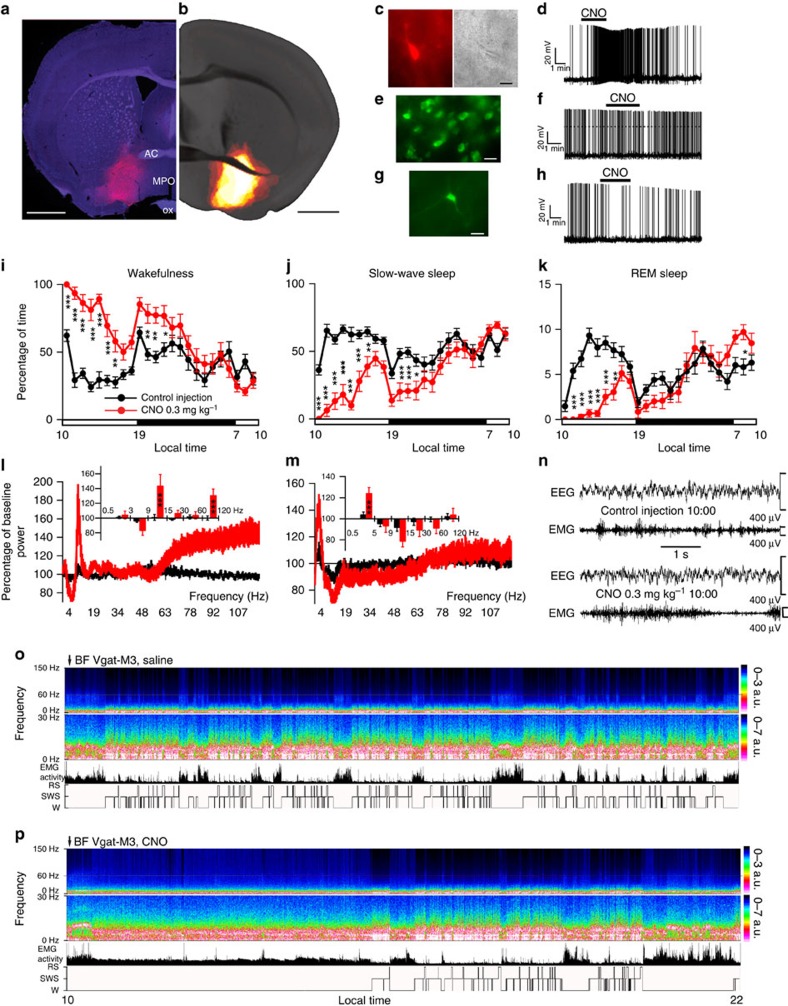
Administration of CNO activates GABAergic BF hM3Dq^+^ neurons, and strongly promoted wake and an increase in fast EEG power density. (**a**) Coronal section from injected *Vgat-IRES-Cre* mouse showing hM3Dq-expressing BF GABAergic (mCherry^+^) neurons (scale bar, 1 mm). (**b**) Heat map generated from ten injection cases in the *Vgat-IRES-Cre* mouse; white colour=area of maximum overlap of hM3Dq-AAV transduction/expression across ten injection cases. (**c**) hM3Dq-expressing BF GABAergic neurons (left) visualized under IR-DIC (right) during whole-cell recordings (scale bar, 20 μm) showed a strong depolarizing and firing response to bath application of CNO (500 nM; (**d**)). (**e**) Non-hM3Dq-expressing GABAergic BF neurons, recorded from *Vgat-IRES-Cre*, *lox-GFP* mice (scale bar, 20 μm) and (**f**) *Vgat-IRES-Cre* mice injected with *hr*GFP-AAV (scale bar, 20 μm) did not respond to CNO (**e**–**h**). (**i**–**k**) Hourly sleep–wake amounts (±s.e.m.) following injection of CNO (0.3 mg kg^−1^, IP, ZT3=1000 hours, *n*=14 mice) or vehicle. Note that activation of BF GABAergic neurons induced a large and sustained increase in wakefulness (W) at the expense of both slow-wave sleep (SWS) and REM sleep (RS). (**l**,**m**) power spectrum changes (±s.e.m.) over baseline during the 3-h post-injection period for vehicle injection as compared with the first hour post-injection period for CNO (0.3 mg kg^−1^, IP, ZT3=1000 hours, *n*=12 mice; (**l**)) or the first hour containing SWS (*n*=9, (**m**)) and the quantitative changes (±s.e.m.) in power for the *δ* (0.5–3 Hz for W and 0.5–5 Hz for SWS), *θ* (3–9 or 5–9 Hz), *α* (9–15 Hz), *β* (15–30 Hz), low *γ* (30–60 Hz) and high *γ* (60–120 Hz) frequency bands following vehicle or CNO administrations. (**n**) EEG/EMG examples from the first hour post-injection show a normal waking state following CNO injection as compared with vehicle injection. (**o**,**p**) Example CSA (0–30 Hz and 0–150 Hz), EMG activity and hypnogram over 12 h following vehicle (**o**) or CNO (0.3 mg kg^−1^, IP; ZT3; (**p**)) administration in a mouse with bilateral hM3Dq receptor expression in BF GABAergic neurons. The power of the fast EEG frequencies, in particular high *γ* (60–150 Hz) was markedly increased as compared with vehicle administration in the same mouse. A black bar (4–6 bins=1–1.5 Hz large) was inserted to patch over the 60 Hz contaminations of the EEG recordings. AC, anterior commissure; BF, basal forebrain; MPO, medial preoptic area; ox,optic chiasm. A two-way analysis of variance using the between-subjects factor of injection (vehicle or CNO) and the within-subjects factors of time of day or frequency band was used to analyse the percentage(s) of time spent in W, SWS and REM sleep or the frequency bands during W and SWS; *P<0.05, **P<0.01 and ***P<0.001; *P*≥0.05=not significant (NS).

Last, we studied the BF GABAergic cell group. Robust cell-surface expression of hM3Dq receptors was observed in the BF of *Vgat-IRES-Cre* mice (Fig. 3a,c) and was absent in non-cre-expressing littermates. Of note, the percentage of neurons transfected by hM3Dq-AAV was less for the GABAergic cell group than for the cholinergic and glutamatergic cell groups (Supplementary Table 3). Similar to BF cholinergic and glutamatergic neurons, we observed the expected depolarization (+5.9±1.0 mV; *n*=9, *P*<0.01 paired *t*-test control versus CNO) and increased firing response to CNO (500 nM), reversal on washout (Fig. 3c,d), again with CNO having no effect on non-hM3Dq-expressing BF GABAergic neurons (resting membrane potential in control: −45.5±3.2; in CNO: −45.5±3.0; *n*=6; *P*=0.99 paired *t*-test; Fig. 3e–h). Injections of CNO also potently drove c-Fos expression in the BF of mice expressing hM3Dq^+^ (Supplementary Table 3 and Supplementary Fig. 5). Behaviourally, following CNO administration at 1000 hours (=ZT3), these mice exhibited a prolonged increase in wakefulness (Fig. 3i,o and Supplementary Table 4). This unexpected increase in waking occurred at the expense of both SWS and REM sleep (Fig. 3j,k and Supplementary Table 4). No behavioural sleep rebound was observed in these mice during the following hours of the dark period (0100 hours to 0700 hours) or the subsequent light period (Fig. 3j,k and Supplementary Table 4), although a highly significant increase in EEG slow-wave activity density, which is a strongly indicative marker of sleep need, was observed during the first hour of SWS that followed CNO-induced wake (Fig. 3m). With respect to the cortical EEG, CNO-induced activation of BF GABAergic neurons produced an increase in higher gamma band activity (*γ*=60–120 Hz) during wakefulness (Fig. 3l,p). The same results were obtained when CNO was administered at the beginning of the dark period (Supplementary Fig. 4b,d,f,h and Supplementary Table 5). Together, the behavioural and EEG findings establish sufficiency of the BF GABAergic group to drive wake and fast cortical rhythms in behaving mice.

### Inhibition and conditional mapping of GABAergic BF neurons

**Figure 4:**
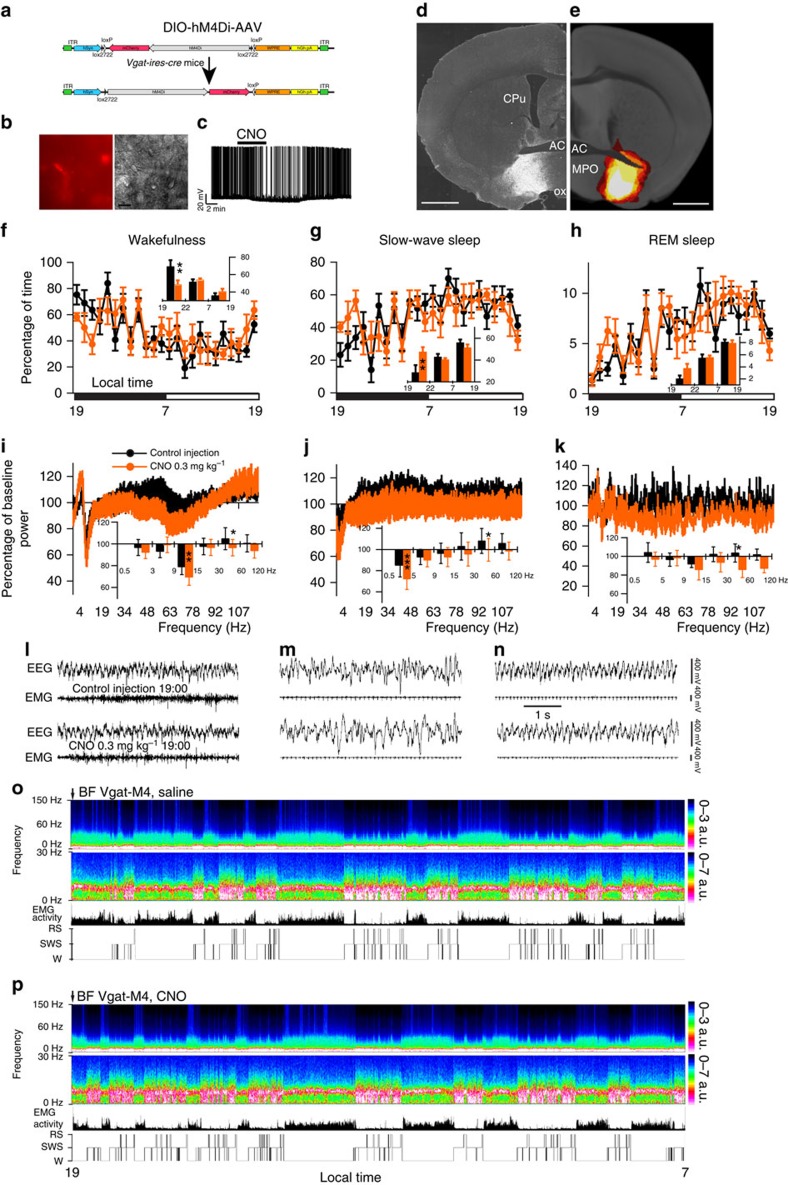
Administration of CNO activates GABAergic BF hM4Di^+^ neurons, and promoted slow-wave sleep (SWS) and an increase in slow EEG power density. (**a**) Experimental design: bilateral injections of DIO-hM4Di-AAV were placed into the BF of *Vgat-IRES-Cre* mice, resulting in expression of hM4Di on BF GABAergic neurons. (**b**,**c**) hM4Di-expressing BF GABAergic neurons (left) visualized under IR-DIC (right) during whole-cell recordings (scale bar, 20 μm) showed a strong membrane hyperpolarization and reduction in firing to bath application of CNO (500 nM; **c**). (**d**) coronal section from injected *Vgat-IRES-Cre* mouse showing hM4Di-expressing BF GABAergic (mCherry^+^) neurons (scale bar, 1 mm). (**e**) Heat map generated from seven injection cases in the Vga*t-IRES-Cre* mouse; white colour=area of maximum overlap of hM4Dq-AAV transduction/expression across seven injection cases. (**f**–**h**) Hourly sleep–wake amounts (±s.e.m.) following injection of CNO (0.3 mg kg^−1^, IP, ZT12=1900 hours, *n*=7 mice) or vehicle; inset in **f** shows ∼30% decrease in wake during the 3-h post CNO injection period. (**i**–**k**) Power spectrum changes (±s.e.m.) over baseline during the 3-h post-injection period for vehicle injection as compared with the 3-h post-injection period for CNO (0.3 mg kg^−1^, IP, ZT3=1900 hours, *n*=5 mice) administration and the quantitative changes (±s.e.m.) in power for the *δ* (0.5–3 Hz for W and 0.5–5 Hz for SWS and REM sleep), *θ* (3–9 or 5–9 Hz), *α* (9–15 Hz), *β* (15–30 Hz), low *γ* (30–60 Hz) and high *γ* (60–120 Hz) frequency bands following vehicle or CNO administrations. (**l**–**n**) EEG/EMG examples of wake (W), SWS and REM sleep (RS) from the first hour post injection of saline (top) or CNO (bottom) in a mouse with bilateral hM4Di receptor expression in BF GABAergic neurons. The raw EEG and EMG traces following CNO injection provide unambiguous evidence of the existence of the three sleep–wake stages while BF GABAergic neurons are inhibited. (**o**,**p**) Example compressed spectral array (CSA; 0–30 Hz and 0–150 Hz), EMG activity and hypnogram over 12 h following vehicle (**o**) or CNO (0.3 mg kg^−1^, IP; ZT12; (**p**)) administration in a mouse with bilateral hM4Di receptor expression in BF GABAergic neurons. A black bar (4–6 bins=1–1.5 Hz large) was inserted to mask the 60 Hz contamination of the EEG recordings. AC, anterior commissure; CPu, caudate putamen; MPO, medial preoptic area; SI, substantia innominata; ox, optic chiasm. A two-way analysis of variance using the between-subjects factor of injection (vehicle or CNO) and the within-subjects factors of time of day or frequency band was used to analyse the percentage(s) of time spent in W, SWS and REM sleep or the frequency bands during W and SWS; *P<0.05, **P<0.01 and ***P<0.001; *P*≥0.05=not significant (NS).

To determine whether BF GABAergic neurons are *necessary* for sustaining normal wakefulness, we expressed a Cre-recombinase-enabled chemogenetic inhibitory (hSyn-DIO-hM4Di-mCherry; Fig. 4a) system, again via AAV injections, in the BF of *Vgat-IRES-Cre* mice. Whole-cell current clamp recordings of BF GABAergic neurons expressing hM4Di showed the expected hyperpolarization (−6.5±1.0 mV; *n*=6, *P*<0.01 paired *t*-test control versus CNO) and decreased firing response to CNO (500 nM), reversal on washout (Fig. 4b,c). Whereas the extent of hM4Di-expressing somata mirrored that seen in the hM3Dq-expressing mice (Fig. 4d,e), CNO administration at 1900 hours (=ZT12) in behaving mice resulted in ∼70% increase in SWS (Fig. 4f inset and Supplementary Table 5) over a 3-h period, indicating that these neurons contribute to the maintenance of normal wakefulness. As compared with controls, no changes were seen in REM sleep or cortical EEG frequency distribution following CNO administration (Fig. 4h–p). Conditional anterograde tracing, with the same injection site and viral serotype revealed the possible structural basis of these behavioural effects, that is, increased wake or sleep, with BF GABA neurons targeting the reticular and midline thalamus, subcortical arousal nodes, and the cerebral cortex (Supplementary Fig. 6a–h). Whether the behavioural and EEG outcomes of our activation and inhibition experiments resulted from the engagement of all GABAergic output circuitries, or rather a single circuit output, such as the thalamus, remains to be clarified. Interestingly, BF GABAergic neurons did not target the sleep-promoting medial or ventrolateral preoptic nuclei, and hence direct inhibition of these forebrain sleep nodes is an unlikely structural basis by which activation of BF GABAergic neurons drive wakefulness.

### Activation of glutamatergic thalamocortical (TC) neurons

**Figure 5:**
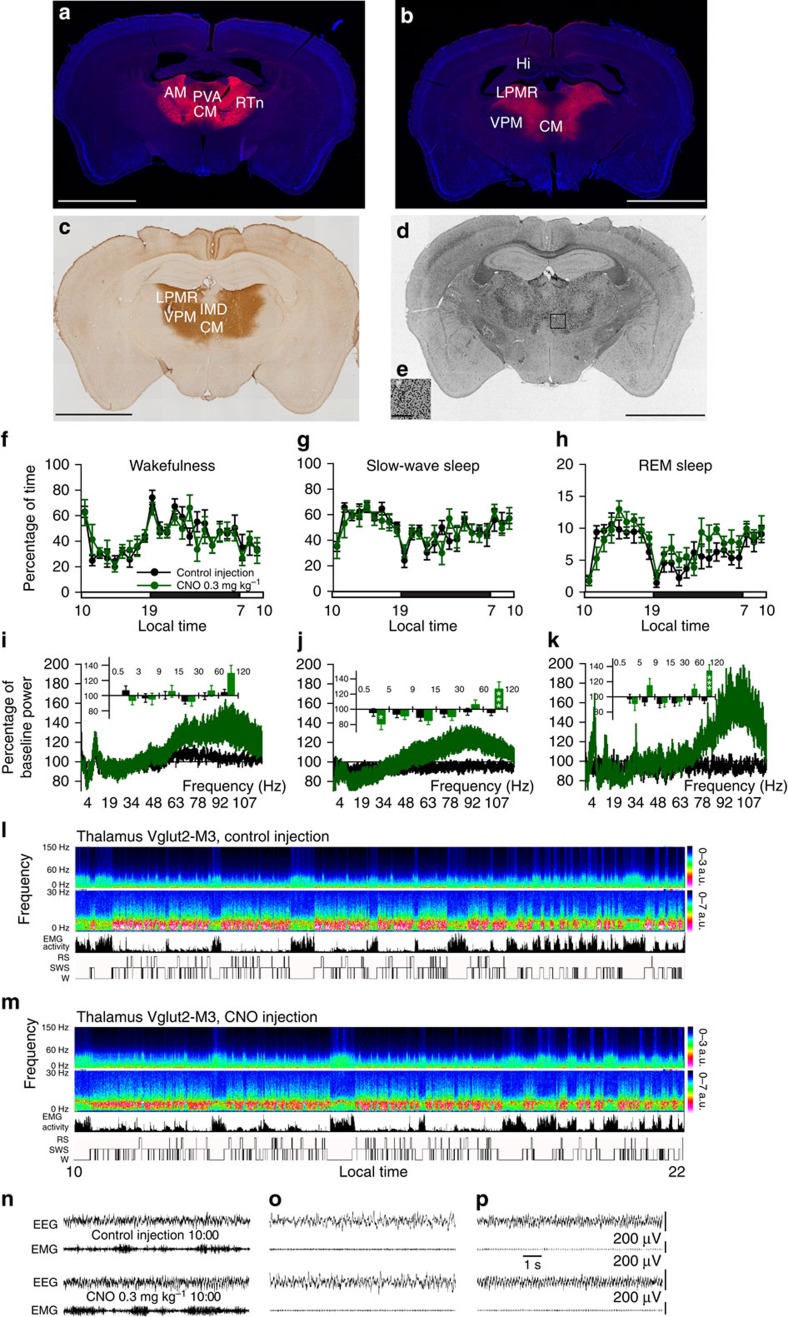
Acute activation of glutamatergic thalamocortical (TC) hM3Dq^+^ neurons did not promote wake, but did increase high-frequency EEG activity during wake, slow-wave sleep (SWS) and REM sleep. Large bilateral injections of hM3Dq-AAV were placed into the thalamus of *Vglut2-IRES-Cre* mice (*n*=8). Rostral and caudal coronal sections from a representative hM3Dq-AAV-injected mouse show widespread expression of hM3Dq in midline and intralaminar TC neurons (**a**–**c**). CNO administration produced robust thalamic c-Fos expression in mice with hM3Dq-AAV injections ((**d**,**e**) corresponding coronal section showing hM3Dq expression in same mouse as panel **a**3). High-power photomicrograph (**e**) of thalamic region in box of **d** shows the robust expression of c-Fos following CNO administration (scale bar, 250 μm). (**f**–**h**) Hourly sleep–wake amounts (±s.e.m.) following injection of CNO (0.3 mg/kg, IP, ZT3=1000 hours, *n*=8 mice) or vehicle. (**i**–**k**) Power spectrum changes (±s.e.m.) over baseline during the 3-h post-injection period for vehicle injection as compared with the 3-h post-injection period for CNO (0.3 mg kg^−1^, IP, ZT3=1000 hours, *n*=8 mice) and the quantitative changes (±s.e.m.) in power for the *δ* (0.5–3 Hz for W and 0.5–5 Hz for SWS and REM sleep), *θ* (3–9 or 5–9 Hz), *α* (9–15 Hz), *β* (15–30 Hz), low *γ* (30–60 Hz) and high *γ* (60–120 Hz) bands following vehicle or CNO administrations. (**l**,**m**) Example CSA (0–30 Hz and 0–150 Hz), EMG activity and hypnogram over 12 h following vehicle (**l**) or CNO (0.3 mg kg^−1^, IP; ZT3; m) administration in a mouse with bilateral hM3Dq receptor expression in thalamus glutamatergic neurons. A black bar (4–6 bins=1–1.5 Hz large) was inserted to mask the 60 Hz contamination of the EEG recordings. (**n**–**p**) EEG/EMG examples of wake (W), SWS and REM sleep (RS) from the first hour post injection of saline (top) or CNO (bottom) in a mouse with bilateral hM3Dq receptor expression in thalamus glutamatergic neurons. The raw EEG and EMG traces following CNO injection provide unambiguous evidence of the existence of the three sleep–wake stages while thalamus glutamatergic neurons are activated. AM, anteriormedial thalamus; CM, centromedial thalamus; Hi, hippocampus; IMD, intermediodorsal thalamus; LPMR, lateral posterior thalamus, mediorostral; PVA, paraventricular thalamus, anterior; RT*n*, reticular thalamus; VPM, ventral thalamus, posteromedial. A two-way analysis of variance using the between-subjects factor of injection (vehicle or CNO) and the within-subjects factors of time of day or frequency band was used to analyse the percentage(s) of time spent in W, SWS and REM sleep or the frequency bands during W and SWS; *P<0.05, **P<0.01 and ***P<0.001; *P*≥0.05=not significant (NS). scale bar (**a**–**d**), 2 mm.

Cortically projecting glutamatergic TC neurons, particularly those of the midline thalamus, have long been considered a critical node of the brain's arousal system^18^,^19^,^20^, yet represent a major postsynaptic target of inhibitory BF GABAergic neurons (Supplementary Fig. 6e,g). Activation of BF GABAergic neurons would therefore *inhibit* this putative thalamic component of the arousal system. To better understand this apparent paradox, and simultaneously test the wake-promoting potential of TC glutamatergic neurons *in vivo*, we placed large bilateral injections of hM3Dq-AAV into the thalamus of *Vglut2-IRES-Cre* mice. This in turn permitted the acute and selective activation of glutamatergic TC neurons, emphasizing those of the midline and intralaminar thalamus, in behaving adult mice. As observed in mice with BF injections of hM3Dq-AAV, hM3Dq receptors were robustly expressed on glutamatergic somata of the midline and intralaminar thalamus (Fig. 5a–c). Moreover, marked c-Fos was noted in the thalamus following CNO administration (Fig. 5d,e), indicating strong activation of TC neurons by the hM3Dq ligand. Unexpectedly, CNO-induced activation of TC neurons did not drive behavioural wake or produce effects on sleep–wake quantity, consolidation or sleep latency (Fig. 5f–h and Supplementary Table 4), although an increase in higher gamma band activity (*γ*=60–120 Hz) was observed across all sleep–wake stages (Fig. 5i–k,m).

## Discussion

A close relationship between the BF and cortical activity has long been appreciated. Direct stimulation of the BF has pronounced activating effects on the cortical EEG^21^, enhances responsiveness to sensory input^5^ and inhibition of BF firing can slow the EEG^22^,^23^. BF neuronal firing is time-locked to EEG oscillations, with changes in cholinergic, glutamatergic and GABAergic firing correlated with specific EEG spectral shifts^16^. BF glutamate infusion increases wake^23^, whereas selective lesions of cholinergic neurons transiently reduce wake, and lesions also including non-cholinergic neurons increase EEG delta activity, reduce wake or result in a low amplitude EEG and behavioural unresponsiveness^9^,^14^. Inhibition of BF neurons with an adenosine A1 agonist promotes sleep, even following lesions of the cholinergic population^24^,^25^. These findings demonstrate an important role for BF cholinergic and non-cholinergic cell groups in the regulation of cortical activity.

BF cholinergic neurons innervate, directly and indirectly, as well as activate, cortical pyramidal cells, and are thought to augment cortical activation and EEG desynchronization^26^,^27^. Behavioural studies in animals and humans have linked damage to the BF cholinergic neurons with cognitive deficits, including impairments in attention, learning and memory processes^1^,^6^,^28^,^29^. Stimulation studies^30^ have reinforced this hypothesis while other work has implicated BF cholinergic neurons in sensory processing and experience-dependent cortical plasticity^4^,^31^,^32^. Degeneration of BF neurons has been observed in a number of neurodegenerative disorders^33^, most notably Alzheimer's disease and dementia with Lewy bodies, and following chronic ethanol intake^34^. Given the foregoing, it is curious that lesions of the BF cholinergic system, even putative ‘cell-specific' lesions, produce only modest, if any, alterations in total wake or EEG responses^9^,^14^. Thus, BF cholinergic neurons may critically augment cognitive processes such as attention, but they may not contribute to the maintenance of the waking state—a fundamental requirement for normal cognition. We show in the present study that acute activation of BF cholinergic neurons influenced both the cortical EEG and the consolidation of wakefulness and sleep, but was without effect on the total time spent awake, or the ability to initiate sleep. Activation of BF cholinergic neurons did, however, destabilize the sleep state, leading to fragmentation. A significant decrease in lower frequency EEG during SWS was also observed after cholinergic neuronal activation, further suggesting that BF cholinergic neurons may function, in part, to suppress lower frequency components of the EEG. In other words, our results suggest that BF cholinergic neurons are not wake-promoting *per se*, but rather may facilitate the EEG correlate of waking by inhibiting EEG slow activity. This interpretation would also be consistent with the sleep-disrupting (that is, increased probability to wake) effects of optogenetic activation of cholinergic neurons during both light^35^ and deep sleep^36^, although we did not observe changes in REM sleep similar to those reported in the latter study^36^. Our findings would therefore predict that the slowing of the cortical EEG and altered state stability, observed in dementias such as Alzheimer's Disease, are due to BF cholinergic cell loss and the concomitant reduction of cholinergic activity.

BF glutamatergic neurons directly innervate cortical interneurons, paralleling projections of BF cholinergic neurons^37^. These (putative) glutamatergic neurons discharge in correlation with fast gamma activity in the EEG, suggesting a possible role in wakefulness^16^. Positive correlation of unit and EMG activity has also been noted, and may link to innervation of lateral hypothalamic hypocretin neurons^16^,^38^. We found that acute activation of BF glutamatergic neurons resulted in a small decrease in EEG delta power during SWS, suggesting a contribution to cortical desynchronization. However, activation was without effect on sleep–wake quantities, had no effect on sleep latency or consolidation, produced no changes in the EMG or EEG fast frequencies, including gamma activity. This lack of an effect on behavioural parameters does not appear to be technical: we saw significant EEG effects as well as robust CNO-induced Fos expression in these neurons (Supplementary Fig. 7a,b), firing response to CNO application *in vitro*, cell transfection was seen in regions known to contain glutamatergic neurons in the BF, and cellular transfection was high (>80%, Supplementary Table 3).

BF GABAergic neurons outnumber cholinergic neurons 2:1 (ref. 39) and exhibit discharge that closely tracks behavioural state, including a wake-active subgroup^15^,^16^. A subpopulation of BF GABAergic neurons directly innervate inhibitory cortical interneurons^40^, which themselves are extensively collateralized, with each contacting hundreds of pyramidal neurons^40^ and providing collaterals to other local BF cells^41^,^42^. Thus, the potential anatomical substrates are in place for BF GABAergic neurons to produce cortical disinhibition. Other subgroups of BF GABAeregic neurons fire maximally during SWS and REM sleep^15^,^16^, suggesting functional heterogeneity within BF GABAergic neurons. Findings from the present study show that acute activation of BF GABAergic neurons, likely comprising all GABAergic subgroups, and including intermingled ventral striatopallidal neurons, potently drives wakefulness and EEG high gamma (60–120 Hz) activity. This wake-promoting effect is unexpected, given that GABAergic neurons are inhibitory and that prevailing models of BF organization have emphasized cholinergic neurons in cortical activation, including fast EEG activity. The present results instead suggest that both the waking state and gamma activity, the latter linked with higher cortical function and conscious awareness^43^, are facilitated by BF GABAergic neuronal activation. As indicated above, histological analysis revealed variable somata expression of hM3Dq within the inferior medial GP in some (6 of 13) *Vgat*-IRES-Cre mice, possibly linking GP activation to the observed waking response. However, all 13 injected *Vgat*-IRES-Cre mice demonstrated significant wake promotion in response to CNO administration, and stimulation and lesion studies have demonstrated a sleep-, not wake-, promoting role for the GP^44^. We moreover show that acute inhibition of BF GABAergic neurons during the early dark period, a typical time of maximal wakefulness in the mouse, resulted in a significant decrease in behavioural wake, demonstrating that BF GABAergic neurons are not only sufficient, but also necessary, for normal wakefulness. It important to recognize that the absolute number of GABAergic neurons in the BF is larger than cholinergic and much larger than the number of glutamatergic neurons, so it is possible that the magnitude of the behavioural and cortical effects were bigger with activation of this larger cell population. However, the key finding that GABAergic neurons are necessary and sufficient for normal wakefulness is robust, and may in fact be due to a subpopulation of GABAergic neurons of unclear absolute number.

Although the circuit basis by which BF GABAergic neurons facilitate wakefulness and fast cortical rhythms remains an open question, our conditional anterograde tracing coupled to the absence of wake-promoting response to thalamic activation indicates that two complementary explanations remain possible: disinhibition of either the cerebral cortex or via sub-cortical, perhaps hypothalamic, components of the arousal system. Confirmation of functional synaptic connectivity between BF GABAergic neurons and their putative postsynaptic targets, which were revealed by the conditional anterograde tracing (Fig. 7a–h) and included the lateral, posterior lateral and supramamillary hypothalamus, reticular thalamus and several cortical areas, remains an area of future investigation. In addition, although activation of all GABAergic neurons of the anatomic BF result in wakefulness, there is good evidence for functional and anatomical subgroups of these neurons. Subgroups of BF GABAergic neurons have been described on the basis of immunostaining for different calcium-binding proteins, such as parvalbumin (PV), calbindin-D28k and calretinin^45^. PV-expressing (PV^+^) BF neurons in particular have received considerable interest as a subgroup of GABAergic neurons, largely due to their projections to the cerebral cortex and reticular thalamus^37^,^46^, but also because they are excited by cholinergic input, possibly from the BF itself^47^. In addition, a recent study found that optogenetic activation of BF PV^+^ neurons elicits cortical gamma band oscillations^48^ (GBOs,∼40 Hz activity), which link to higher cognitive function; effects of BF PV^+^ neuronal activation on wakefulness were not reported. In the present study, activation of GABAergic BF neurons produced high gamma EEG activity, but did not specifically elicit GBO, suggesting that either concurrent activation of non-PV^+^ and PV^+^ GABAergic neurons had a suppressive effect on GBO generation or that the expression of GBO requires the precise stimulation paradigm used in the optogenetic study. Alternatively, because BF PV^+^ neurons are not exclusively GABAergic^45^,^49^, their selective activation may have produced both excitatory and inhibitory postsynaptic responses in downstream targets, including within the cortex, to produce GBO. It should finally be acknowledged that even though our MRIcron-derived colour maps (shown in Figs 1c, 2b, 3b and 4e) define the overlapping areas containing transfected soma for the injection cases, future work using smaller injection volumes will be required to delimit the precise anatomic subregion—and possibly functional subgroup—comprising the wake-promoting GABAergic BF.

Although activation of GABAergic neurons of the BF potently promoted prolonged arousal, similar activation of glutamatergic neurons of the midline and intralaminar thalamus, one major postsynaptic target of BF GABAergic neurons, did not drive wakefulness. The purpose of this inhibitory projection from the BF to the thalamus thus remains unclear, but it seems unlikely that the BF exerts its wake-promoting effect via this thalamic projection or that thalamic activity is required for a normal *level* of wakefulness. Our findings, and numerous others, instead suggest a critical role for the thalamus in cognition and cortical function^50^, but not for the *level* of behavioural or EEG arousal, or organization of the sleep–wake cycle^9^,^51^,^52^, including in humans^53^,^54^. However, the BF, and specifically GABAergic BF neurons, appear to play a key and under-appreciated role in regulating the level of wakefulness/arousal.

In summary, the present study shows for the first time that activation of GABAergic BF neurons facilitates wakefulness and high-frequency EEG activity in behaving animals, and that they are necessary for normal levels of wakefulness. In contrast, cholinergic and glutamatergic neurons of the BF appear to help suppress cortical slowing, whereas glutamatergic TC neurons, although critical for cortical function, do not contribute meaningfully to the level of wakefulness. These findings suggest that BF GABAergic neurons may form the structural basis for a generator of EEG gamma activity and are likely necessary for normal conscious awareness, at least in part through interaction with, or in addition to, fast-spiking (PV-expressing) cortical interneurons^55^. As such, the BF, and its GABAergic neurons in particular, warrants consideration as a stimulation target for patients with disorders of consciousness. In fact, non-selective BF deep brain stimulation, with appropriately chosen parameters that do not result in inhibition, given that glutamate and acetylcholine neurons would not antagonize the wake-promoting effects of GABAergic neuronal activation, should be considered in selected patients with impairments of consciousness. Although thalamic-based deep brain stimulation has been used to increase behavioural responsiveness in patients in the minimally conscious state^56^, our present findings suggest that stimulation of the BF may have a more potent facilitating effect on the level of wakefulness.

## Methods

### Animals

Adult male *ChAT-IRES-cre*^57^, *Vglut2-IRES-cre*^58^ and *Vgat-IRES-Cre*^59^ mice and non-cre-expressing littermate mice (8–12 weeks, 20–25 g; *n*=86 *in vivo* and *n*=24 *in vitro*) were used in this study. In all three lines of mice, Cre recombinase was targeted just distal to the stop codon of the *VGAT*, *VGLUT2* or *ChAT* genes, respectively, using an optimized internal ribosomal entry sequence (IRES), hence endogenous gene promoters drove Cre recombinase expression via a bicistronic message. All mice were bred at our animal facility and underwent genotyping both before and after experiments and all procedures were approved by the Institutional Animal Care and Use Committee of Beth Israel Deaconess Medical Center.

### Mouse validation

As previously done, all mouse lines were first crossed to a reporter, in this case the L10 GFP^59^ reporter, to confirm that Cre activity mapped to sites where the respective neuronal subtypes are located as well as the extent of ectopic expression, if any. We observed some ectopic expression in the *Vglut2-IRES-Cre* and *ChAT-IRES-Cre* mice. In the case of the Vglut2-cre mouse, the ectopic expression was largely outside of the BF (for example, cortex and hippocampus), whereas in the case of the *ChAT-IRES-Cre* mice, ectopic expression was noted in the BF. As we could not ascertain whether the reporter expression (that is, GFP^+^ cells) was related to transient developmental promoter activity or represented true ectopic expression in the adult mouse, we performed either dual immunohistochemical analysis or combined immunohistochemical and *in situ* hybridization analysis on tissue from each mouse line with AAV-based injections of cre-dependent mCherry targeting the BF. Using this approach, we observed virtually no ectopic expression of mCherry within the BF of any of the mouse lines, strongly suggesting that the ectopic expression observed in the reporter crosses links to transient promoter activity during the development. Details of the tissue processing are described below.

### Surgery

Mice were anaesthetized with ketamine/xylazine (100 and 10 mg kg^−1^, respectively, IP) and then placed in a stereotaxic apparatus. To selectively express the hM3Dq receptors in cholinergic (ChAT^+^), glutamatergic (VGLUT2^+^) or GABAergic (VGAT^+^) neurons of the BF, we placed bilateral injections of an AAV (serotype 10; 60 nl per side) vector expressing the hM3Dq receptor in a cre-dependent configuration (hSyn-DIO-hM3Dq-mCherry-AAV; hM3Dq-AAV) into the BF (coordinates: anterioposterior (AP)=−0.2 mm, mediolateral (ML)=±0.1 mm, dorsoventral (DV)=−4.6 mm, as per the mouse atlas of Paxinos and Watson) of *Vgat-IRES-Cre* (*n*=13), *Vglut2-IRES-Cre* (*n*=11) and *ChAT-IRES-Cre* mice (*n*=14). As vector injection controls, we injected hM3Dq-AAV into the BF of non-cre-expressing littermate mice (*n*=8, 9 and 7, respectively). To selectively express the hM4Di receptors in GABAergic (VGAT^+^) neurons of the magnocellular BF, we placed bilateral injections of an AAV (serotype 10) vector expressing the hM4Di receptor in a cre-dependent configuration (hSyn-DIO-hM4Di-mCherry-AAV; hM4Di-AAV) into the BF of *Vgat-IRES-Cre* (*n*=7). Injections of hM3Dq-AAV or hM4Di (serotype 10; 60 nl per side) into the BF of these mice were performed using a compressed air delivery system as previously described^17^. To selectively express the hM3Dq receptor in glutamatergic TC neurons, we placed bilateral injections of an AAV vector encoding hM3Dq-AAV (serotype 10; 350 nl per side) into the thalamus (coordinates: AP=−0.95 mm, *L*=±0.7 mm, DV=−3.3 mm, as per the mouse atlas of Paxinos and Watson) of *Vglut2-IRES-Cre* mice or non-cre-expressing littermates (hM3Dq *n*=8). After injections, mice were implanted with four EEG screw electrodes (two frontal and two parietal electrodes; Pinnacle Technology Inc.) and two flexible EMG wire electrodes (Plastics One) previously soldered to a 6-pin connector (Heilind Electronics, Inc.) and the assembly was secured with dental cement. The scalp wound was closed with surgical sutures and the mouse was kept in a warm environment until resuming normal activity as previously described^17^. The frontal electrodes were positioned 1 mm frontal and 1 mm lateral of bregma (Note: this region of the cortex is densely innervated by thalamic regions containing TC neuron that were activated in our study). The parietal electrodes were positioned 1 mm lateral from bregma and midway between bregma and lambda.

### Sleep–wake monitoring

Three weeks after surgery, the mice were housed individually in transparent barrels in an insulated sound-proofed recording chamber maintained at an ambient temperature of 22±1 °C and on a 12-h light/dark cycle (lights-on at 0700 hours, Zeitgeber time: ZT0) with food and water available *ad libitum*. Mice were habituated to the recording cable for 5–7 days before starting polygraphic recording. Cortical EEG (ipsilateral frontoparietal leads) and EMG signals were amplified and digitalized with a resolution of 500 Hz using Vital recorder (Kissei). Mice were recorded for 24 h baseline period followed by injections of CNO (Sigma-Aldrich; 0.3 mg kg^−1^ in saline, IP) injections at 1000 hours (ZT3, light period, time of high sleeping drive) and 1900 hours (ZT12, lights-off, time of high activity). As an injection control mice were injected with saline at 1000 hours and 1900 hours CNO and saline injections were performed in a random sequence and separated by 3–5 days washout period. The injections were performed using a cross-over design.

### hM3Dq/hM4Di-AAV and CNO

For the *in vivo* studies, we employed evolved G protein–coupled muscarinic receptors (hM3Dq and hM4Di) that are selectively activated by the pharmacologically inert drug CNO. This system was first developed and described by the Roth laboratory^60^. In our studies, cre-dependent versions of the hM3Dq and hM4Di receptors were packaged into an AAV to facilitate the stereotaxic-based delivery and regionally restricted expression of hM3Dq and hM4Di. As demonstrated previously by our lab^17^ and others and additionally confirmed by using sleep–wake parameters—for all three cre driver mouse lines—in the present study, both the hM3Dq/hM4Di receptor and ligand are biologically inert in isolation (Supplementary Figs 2 and 3).

### Sleep scoring and analysis

Using SleepSign for Animal (Kissei) and with assistance of spectral analysis using fast Fourier transform, polygraphic records were visually scored by 10 s epochs for wakefulness (W), SWS and REM sleep^17^. The percentage of time spent in W, SWS and REM sleep, as well as the number and the average durations of the episode was summarized for each group and each condition. The latency to SWS and REM sleep is defined as the time between the end of the injection and the onset of the first SWS episode lasting >20 s and the first REM sleep episode lasting >10 s.

Recordings were scored again in 5 s epochs to allow for performance of an EEG power spectrum analysis. On the basis of visual and spectral analysis, epochs containing artefacts occurring during active wake (with large movements) or containing two vigilance states were visually identified and omitted from the spectral analysis. Recordings containing wake artefact during more than 20% of the time were removed from the spectral analysis. EEG power spectra were computed for consecutive 5 s epochs within the frequency range of 0.5–120 Hz using a fast Fourier transform routine. The data were collapsed into 0.5 Hz bins. The data were standardized by either expressing each frequency bin as a percentage relative to the total power (for example, bin power/0.5–120 Hz total power; Supplementary Fig. 2) of the same epochs or expressing each frequency bin as a percentage relative to the same bin in baseline condition from the same mouse and from the same time of the day (same Zeitgeber time; Figs 1, 2, 3, 4, 5 and Supplementary Figs 3 and 4). To analyse the EEG frequency bands, relative power bins were summed in *δ*=0.5–3 Hz or 0.5–5 Hz, *θ*=3–9 Hz or 5–9 Hz, *α*=9–15 Hz, *β*=15–30 Hz, low *γ*=30–60 Hz and high *γ*=60–120 Hz.

The individuals performing the saline/CNO injections and sleep–wake analysis did not perform the genotyping or initial immunohistochemical assessment of the injection sites.

### Spectral analysis—compressed spectral array (CSA)

SleepSign files were converted to European Data Format (EDF) and groups of channels for each mouse were further separated into EDF files with Sirenia Sleep (Pinnacle Technologies). Each EDF file was then opened in Persyst Insight II and Magic Marker (v1.1, PersystZ) and then converted into a CSA using Fourier Analysis (500 Hz data, 2,048 points Fourier transform routine, 5 overlapping windows, notch filter 60 Hz) into 10.24 s epoch with spectral resolution of 0.2441 Hz. CSAs were created for the ranges 0–150 and 0–30 Hz, 12 h in length. An amplitude bar graph was simultaneously created as the average of 10.24 s epoch also. Bit maps were exported with full resolution (4,219 pixels) in TIFF format for the creation of figures. The CSA spectral power, with range indicated by the ribbon in each figure, is expressed as arbitrary units, kept constant for each mouse between saline and CNO conditions, corresponding to a scale of about 0–3 μV Hz^−1^ for 0–150 Hz panels and 0–7 μV Hz^−1^ for 0–30 Hz panels. EMG amplitude was kept constant for a given mouse and is typically from 0 to 150 μV. Notch filtering of line frequency noise resulted in significant roll-off, so 60 Hz activity was instead masked graphically in CSA figures.

### Statistical analysis

Statistical analysis was performed using Prism v6 (GraphPad Software). Following confirmation that the data met the assumptions of the analysis of variance model, a two-way analysis of variance followed by a *post hoc* Bonferroni test were used to compare the effects of the genotype or the drug injection on sleep–wake parameters. Paired *t*-tests were used to analyse the latency to SWS or REM sleep. Sample size and power calculations were performed *post hoc* at http://www.biomath.info, using means and standard deviations derived from our analysis. The present study was sufficiently powered to detect effect sizes.

### Plotting of injection sites as ‘heat-maps'

A standard mouse brain MRI^61^ was resampled and rotated (Mango, Research Imaging Institute, University of Text Health Science Center) to match the slice thickness and plane of section of the above histological sections. Injection sites were viewed under both high and low power to discern the region of transduction of somata, and an outline containing this region was then drawn on the corresponding MRI section using MRICron (Rorden and Colleagues, McCausland Center for Brain Imaging, University of South Carolina). A colour map was chosen to show the region of overlap of the maximum number of overlapping cases in white, with the hues to yellow, orange, to deep red indicating fewer cases.

### Immunohistochemistry

Animals received CNO (0.3 mg kg^−1^, IP 1000 hours) and were killed 2 h later by deep anaesthesia with 200 mg kg^−1^ of chloral hydrate, followed by transcardial perfusion with 20 ml saline, followed by 100 ml of neutral phosphate-buffered formalin (4%, Fischer Scientific Co.). Brains were removed, incubated in 20% sucrose at 4 °C until they sank, and then sectioned at 40 μm on a freezing microtome in three series. For all c-Fos and DsRed immunohistochemical staining that involved visualization using a diaminobenzidine reaction, the sections were incubated overnight with primary antiserum (1:20 K, c-Fos; 1:10 K DsRed; 1:2 K ChAT) and then incubated in the respective secondary antibodies for 2 h, followed by incubation in ABC reagents (1:1,000; Vector Laboratories) for 90 min, then washed again and incubated in a 0.06% solution of 3,3-diaminobenzidine tetrahydrochloride (Sigma-Aldrich) or 0.06% solution of 3,3-diaminobenzidine tetrahydrochloride and 0.05% CoCl_2_ and 0.01% NiSO_4_ (NH_4_) in PBS plus 0.02% H_2_O_2_ for 5 min. Finally, the sections were mounted on slides, dehydrated, cleared and coverslipped. Sections for immunofluorescence staining were incubated in fluorescent-labelled secondary antibody for 2 h and coverslipped with DAPI-infused fluorescence mounting media.

### Antibody characterization

The rabbit polyclonal Fos antibody was raised against a synthetic peptide including residues 4–17 from human c-Fos (Oncogene Sciences, rabbit polyclonal cat # Ab5; note that this Ab is not longer commercially available). This antibody stained a single band of 55 kDa m.w. on western blots from rat brain (manufacturer's technical information). c-Fos staining with the Ab5 antiserum is found in many CNS structures only when neurons within these structures have recently been physiologically stimulated.

The rabbit polyclonal antibody against mCherry was raised against DsRed (Clontech, cat#632496) and the specificity of immunostaining for DsRed was indicated by the lack of detectable immunostaining in uninjected mice.

The goat polyclonal antibody (Chemicon, AB144) against ChAT was raised against human placental enzyme, and only a single band of appropriate molecular weight was identified by immunoblot in mouse NIH/3T3 cell lysates (manufacturer's technical information). In the BF and the mesopontine tegmentum, it stains a pattern of cellular morphology and distribution identical to that of previous reports.

For all secondary antibody immunohistochemical controls, the primary antibodies were omitted and the tissue showed no immunoreactivity above background.

Secondary antibodies included: Donkey anti-rabbit Alexa fluor 546 (Invitrogen; 1:500) and Donkey anti-rabbit biotinylated (Invitrogen; 1:500).

### *In situ* hybridization

As indicated above, some ectopic expression was noted in our GFP reporter crosses, in particular for the *Vglut2-IRES-Cre* and *ChAT-IRES-Cre* mouse lines, and so we sought further histological confirmation that our cre-dependent expression cassettes were expressed exclusively in ChAT, Vglut2 or Vgat BF neurons of the *ChAT-, Vglut2*- and *Vgat-IRES-cre* mice, respectively. To do this, we performed dual immunohistochemical staining for mCherry and ChAT in our *ChAT-IRES-cre* mice or combined immunohistochemical and *in situ* hybridization for mCherry and Vgat or Vglut2, respectively, in our *Vgat-IRES-cre* and *Vglut2-IRES-cre* mice. For the combined immunohistochemical and *in situ* hybridization work, free-floating sections were incubated overnight with hybridization buffer containing probes for VGLUT2 or VGAT at 53 °C. Sections were then rinsed in 2 × standard saline citrate with 50% formamide (2 × 1 h) and in 1 × standard saline citrate with 50% formamide (3 × 1 h) at 53 °C. After rinsing in Tris-buffered saline (TBS) pH 7.5 (3 × 10 min), the sections were incubated for 30 min in 1% H_2_O_2_ TBS (pH 7.5). After additional, thorough, TBS rinses (3 × 10 min), the sections were incubated for 30 min in 1% blocking reagent (Roche, cat # 11 096 176 001) and incubated overnight in peroxidise-conjugated anti-digoxigenin antibody (1:500; Roche cat # 11 207 733 910). Sections were rinsed in TBS (3 × 10 min) and then reacted with 1:50 Tyramide signal amplification Dinitrophenyl (TSA Plus-DNP, Perkin Elmer, cat # NEL747B001KT) or 1:50 TS Plus-Cy5 (Perkin Elmer, cat # NEL745001KT) diluted in amplification diluent (30 min). After rinsing in TBS (10 min) and PBS (3 × 5 min), sections were incubated in 1:5,000 rabbit anti-Ds-Red (Clontech, 632946) overnight at room temperature, and then reacted with the secondary antibody to rabbit IgG (Cy3-conjugated Dk α Rb-Fab fragments, Jackson ImmunoResearch, 711-167-003) for 2 h. After rinsing in PBS (three times for 10 min), the sections were incubated in 1:500 Alexa488-conjugated anti-DNP in 0.2% Triton-PBS (3 h, Molecular Probes cat # A11097). Finally, sections were washed in PBS, mounted on slides and coverslipped in PBS for imaging. The VGAT and VGLUT2 probes were produced by Dr Shigefumi Yokota, University School of Medicine, Izumo, Japan.

### Packaging of DIO-hM3Dq and DIO-hM4D AAVs

We used the pAAV-hSyn-DIO-hM3D-mCherry and pAAV-hSyn-DIO-hM4D-mCherry plasmids (gift from B. Roth) to provide the Cre-dependent hM3D transgene for packaging into AAV2/10. Packaging was carried out using a standard triple transfection protocol to generate helper virus-free pseudotyped AAV2/10 virus. An AAV 2/10 rep/cap plasmid provided AAV2 replicase and AAV10 capsid functions, whereas adenoviral helper functions were supplied by the pHelper plasmid (Stratagene). Briefly, AAV-293 cells were transfected via calcium phosphate precipitation with 1.33 pmol of pHelper, and 1.15 pmol each of AAV2/10 and an AAV vector plasmid (double-floxed). The cells were harvested 72 h later and the pellets resuspended in DMEM, freeze-thawed three times and centrifuged to produce a clarified viral lysate. The vector stocks were titred by real-time PCR using an Eppendorf Realplex machine as previously described. The titre of the preparations ranged from ∼1 × 10^12^ to 1 × 10 vector genomes copies per ml^62^. Before initiating the *in vivo* experiments, an absolute requirement for Cre-enabled expression of hM3Dq and hM4Di was confirmed *in vitro* using 293 cre cells.

### Whole cell *in vitro* experiments

*ChAT-IRES-cre* (*n*=6), *Vglut2-IRES-Cre* (*n*=9), *Vgat-IRES-Cre* (*n*=8) and *Vgat-IRES-Cre*, lox-GFP (*n*=1) mice (8 weeks, 20–25 g) were used for *in vitro* electrophysiological recordings. Unilateral injections (30 nl) of hM3Dq-AAV, hM4Di-AAV or *Renilla* Green Fluorescent Protein (hrGFP) hrGFP-AAV (control injections) were placed into the BF of *ChAT-IRES-cre*, *Vgat-IRES-Cre* and *Vglut2-IRES-Cre* mice. Given our finding that the reporter crosses were potentially confounded on the basis of established (developmental) ectopic expression, in particular for the *ChAT-IRES-cre* mouse line, we used hrGFP-injected mice for recording controls. Four weeks after AAV injections, the mice used to prepare BF slices for electrophysiological recordings. Mice were anaesthetized with isoflurane (inhalation, 5% in oxygen) and transcardially perfused with ice-cold artificial cerebral spinal fluid (ACSF;*N*-methyl-D-glucamine, NMDG-based solution) containing (in mM): 100 NMDG, 2.5 KCl, 20 HEPES, 1.24 NaH_2_PO_4_, 30 NaHCO_3_, 25 glucose, 2 thiourea, 5 L-ascorbic acid, 3 Na-pyruvate, 0.5 CaCl_2_, 10 MgSO_4_ (pH 7.3 with HCl when carbogenated with 95% O_2_ and 5% CO_2_). Their brains were quickly removed and cut in coronal brain slices (250 μm thick) in ice-cold NMDG-based ACSF using a vibrating microtome (VT10005, Leica). Slices containing the BF were transferred to normal ACSF containing (in mM): 120 NaCl, 2.5 KCl, 10 glucose, 26 NaHCO_3_, 1.24 NaH_2_PO_4_, 4 CaCl_2_, 1.3 MgCl_2_, 2 thiourea, 1 L-ascorbic acid, 3 Na-pyruvate (pH 7.4 when carbogenated with 95% O_2_ and 5% CO_2_, 310–320 mOsm). Recordings were guided using a combination of fluorescence and infrared differential interference contrast (IR-DIC) video microscopy using a fixed stage upright microscope (BX51WI, Olympus America Inc.) equipped with a Nomarski water immersion lens (× 40/0.8 W) and IR-sensitive CCD camera (ORCA-ER, Hamamatsu) and images were displayed on a computer screen in real time using AxioVision software (Carl Zeiss MicroImaging). Recordings were conducted in whole-cell configuration using a Multiclamp 700B amplifier (Molecular Devices), a Digidata 1300A interface and Clampex 9.0 software (Molecular Devices). Recordings were done using a pipette solution containing (in mM): 120 K-gluconate, 10 KCl, 3 MgCl_2_, 10 HEPES, 2.5 K-ATP, 0.5 Na-GTP and 0.5% biocytin or 0.1% Lucifer yellow CH ammonium salt (0.1%) was added to the pipette solution to label the recorded cells (pH adjusted to 7.2 with KOH, 280 mOsm). For analysis group, means were compared using paired *t*-tests.

## Additional information

**How to cite this article:** Anaclet, C. *et al.* Basal forebrain control of wakefulness and cortical rhythms. *Nat. Commun.* 6:8744 doi: 10.1038/ncomms9744 (2015).

## Supplementary Material

Supplementary Information Supplementary Figures 1-7 and Supplementary Tables 1-5
