# Supplementary material for: Basal forebrain control of wakefulness and cortical rhythms
Source: Nat Commun. 2015 Nov 3;6:8744. doi: 10.1038/ncomms9744 (PMC4659943; doi:10.1038/ncomms9744)
Supplement: Supplementary Information — Supplementary Figures 1-7 and Supplementary Tables 1-5 [file ncomms9744-s1.pdf]

SUPPLEMENTARY INFORMATION
Supplementary Figures

Supplementary Figure 1

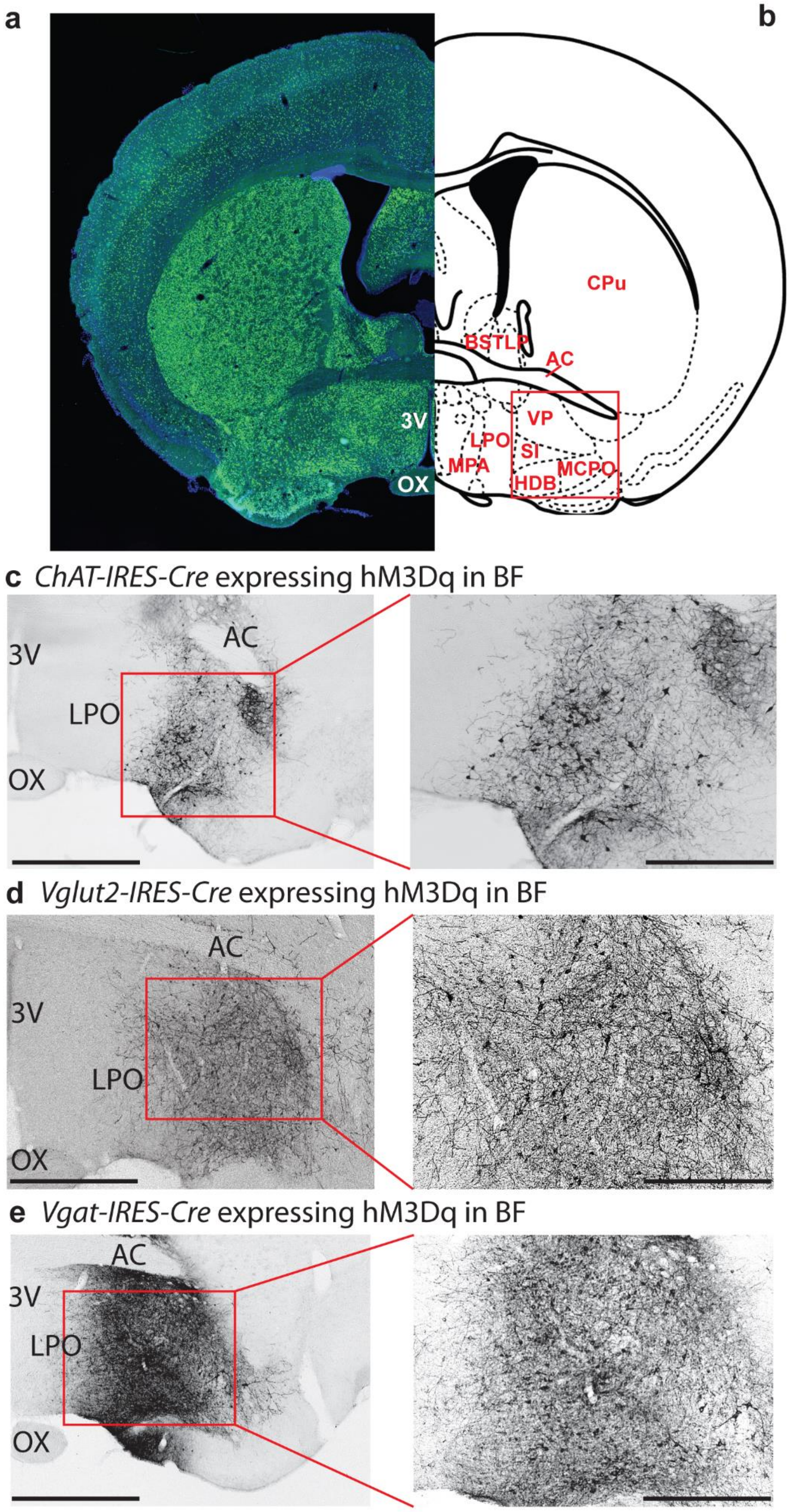

Supplementary Figure 1. Representative hM3Dq-AAV injection cases into the basal forebrain (BF) of Chat-IRES-Cre, Vglut2-IRES-Cre and Vgat-IRES-cre mice. (**a**) Coronal section from a Vgat-IRES-cre, lox-GFP (L10) mouse (**b**) corresponding atlas-derived coronal section showing the BF region targeted in our study (red box). Equal volume injections of hM3Dq-AAV produced strong somata and neuropil labeling in the BF of (**c**) ChAT-, (**d**) Vglut2- and (**e**) Vgat-IRES-cre mice, respectively. The slightly different distribution pattern of transfected somata as well as somata size within the BF between mouse lines appropriately reflects the fact that cre-enabled expression of hM3Dq was dependent upon the endogenous gene promoters driving bicistronic expression of Cre recombinase.

Supplementary Figure 2

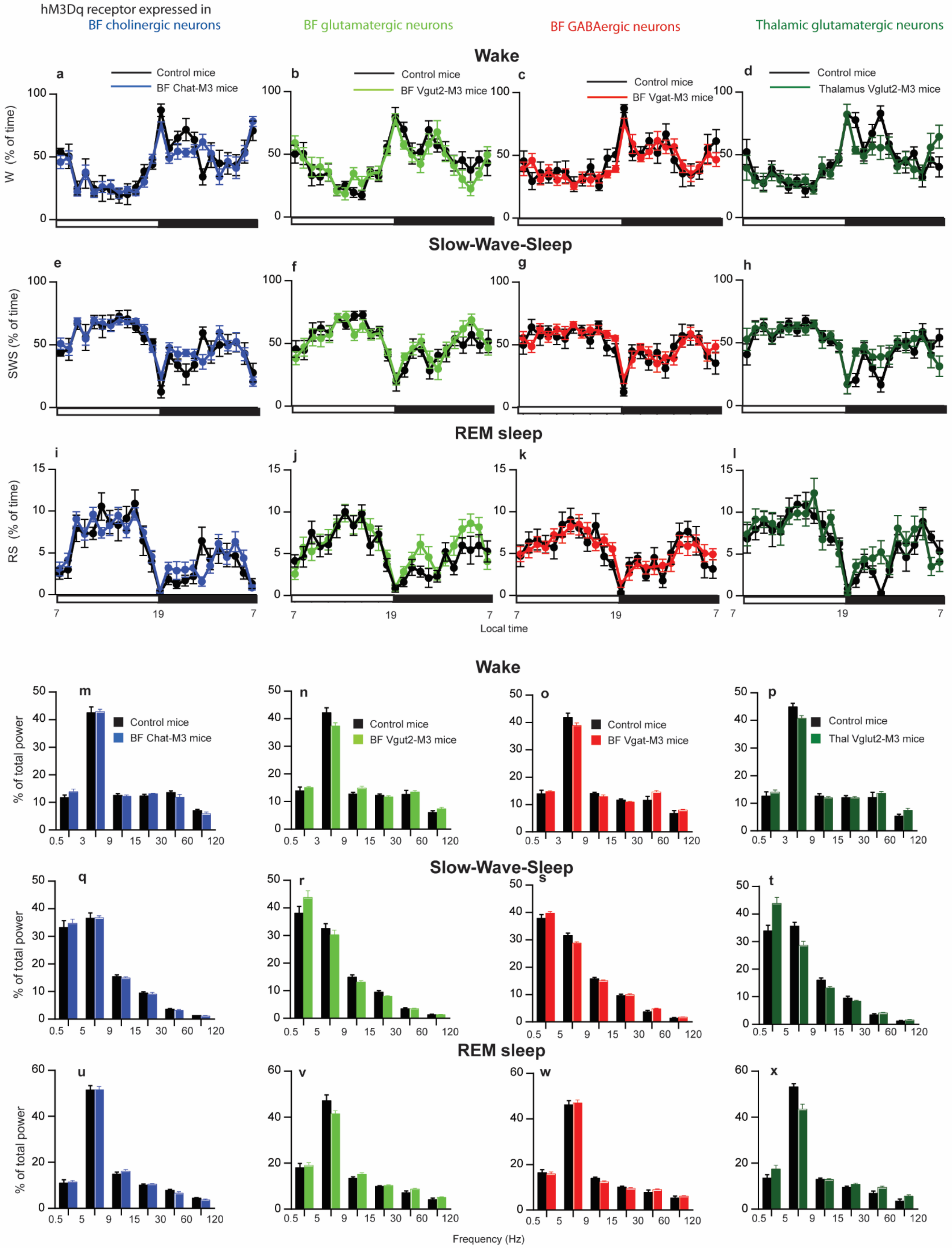

Supplementary Figure 2. In the absence of CNO, hM3Dq receptor expression in basal forebrain (BF) or thalamocortical (TC) neurons was without effect on the sleep-wake cycle or EEG power distribution.

(**a-l**) Hourly sleep-wake amounts (±s.e.m.) in baseline condition of mice expressing hM3Dq receptor in BF cholinergic (**blue**; n = 14), glutamatergic (**light green**; n = 11) or GABAergic (**red**; n= 13) neurons, and in glutamatergic TC neurons (**dark green**; n = 8) as compared with non-cre-expressing littermate mice (n = 7, 9, 8 and 8 respectively). Sleep-wake amounts were not affected by the expression of hM3Dq receptor by any of these neuronal type, indicating that in absence of its ligand, CNO, hM3Dq receptor have no effect on sleep, wake and REM amounts. A two-way ANOVA using the between-subjects factor of hM3Dq expression (control and BF Vgat-, ChAT- or Vglut2-hM3Dq, or thalamus Vglut2-hM3Dq expressing mice) and the within-subjects factors of time of day (hourly) was used to analyze the percentage(s) of time spent in W, SWS and REM sleep. No significance was found. (**m-x**) Power density (±s.e.m.) for the δ (0.5-3 or 0.5-5 Hz), θ (3-9 or 5-9 Hz), α (9-15 Hz), β (15-30 Hz), low γ (30-60 Hz) and high γ (60-120 Hz) frequency bands in baseline condition of mice expressing hM3Dq receptor in BF cholinergic (**blue**; n = 14), glutamatergic (**light green**; n = 10) or GABAergic (**red**; n= 11) neurons, and in glutamatergic TC neurons (**dark green**; n = 7) as compared with non-cre-expressing littermate mice (n = 7, 9, 8 and 7 respectively). . Sleep-wake power bands were not affected by the expression of hM3Dq receptor by any of these neuronal types, indicating that in absence of its ligand, CNO, hM3Dq receptors have no effect on cortical EEG. A two-way ANOVA using the between-subjects factor of hM3Dq expression (control and BF Vgat-, ChAT- or Vglut2-hM3Dq, or thalamus Vglut2-hM3Dq expressing mice) and the within-subjects factors of power bands was used to analyze the EEG power in W, SWS and REM sleep. No significance differences were revealed.

Supplementary Figure 3

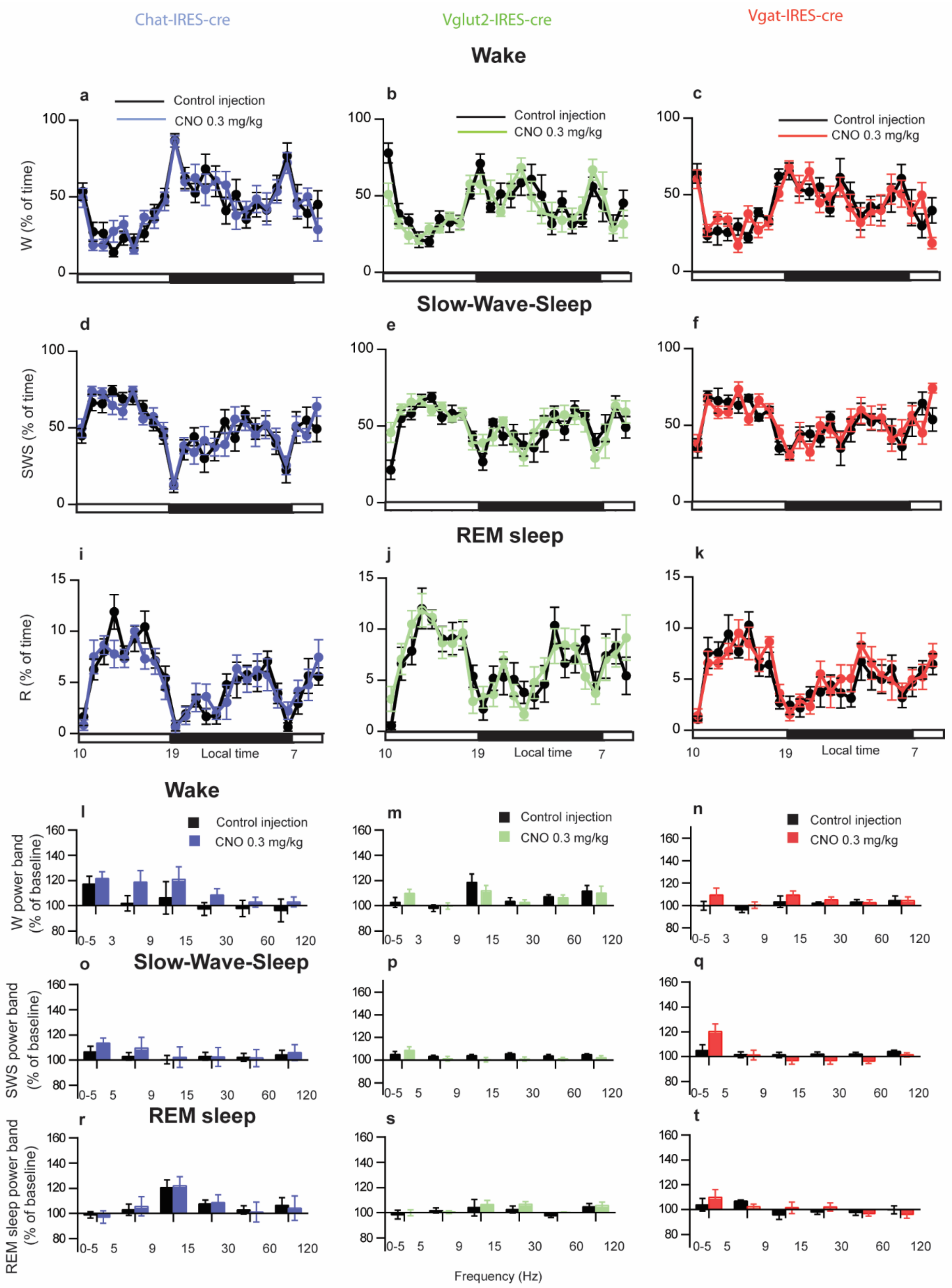

Supplementary Figure 3. CNO was without effect on sleep-wake or EEG power distribution in non-hM3Dq-expressing ChAT-, Vgat- and Vglut2-IRES-cre littermates.

(**a-i**) Hourly sleep-wake amounts (± SEM) following control or CNO (0.3 mg/kg, IP, ZT3) injections in non-hM3Dq-expressing littermates in cholinergic (**blue**, n = 7), glutamatergic (**green**, n = 9) or GABAergic (**red**, n= 8) neurons. Sleep-wake amounts were not affected by the CNO injection in any of these mouse lines, indicating that in absence of its ligand target, the hM3Dq receptor, CNO injection has no effect on sleep, wake and REM amounts. A two-way ANOVA using the between-subjects factor of injection (control and CNO injection) and the within-subjects factors of time of day (hourly) was used to analyze the percentage(s) of time spent in W, SWS and REM sleep. No significance was found. (**j-r**) Power density for the δ (0.5-3 or 0.5-5 Hz), θ (3-9 or 5-9 Hz), α (9-15 Hz), β (15-30 Hz), low γ (30-60 Hz) and high γ (60-120 Hz) frequency bands following control or CNO (0.3 mg/kg, IP, ZT3) injections in mice in non-hM3Dq-expressing littermates in cholinergic (**blue**; n = 7), glutamatergic (**green**; n = 7) or GABAergic (**red**; n= 8) neurons. A two-way ANOVA using the between-subjects factor of injection (control and CNO injection) and the within-subjects factors of power bands was used to analyze the EEG power in W, SWS and REM sleep. No significance was found, indicating that in absence of its ligand, CNO, expression of hM3Dq receptors is without effect on cortical EEG.

Supplementary Figure 4

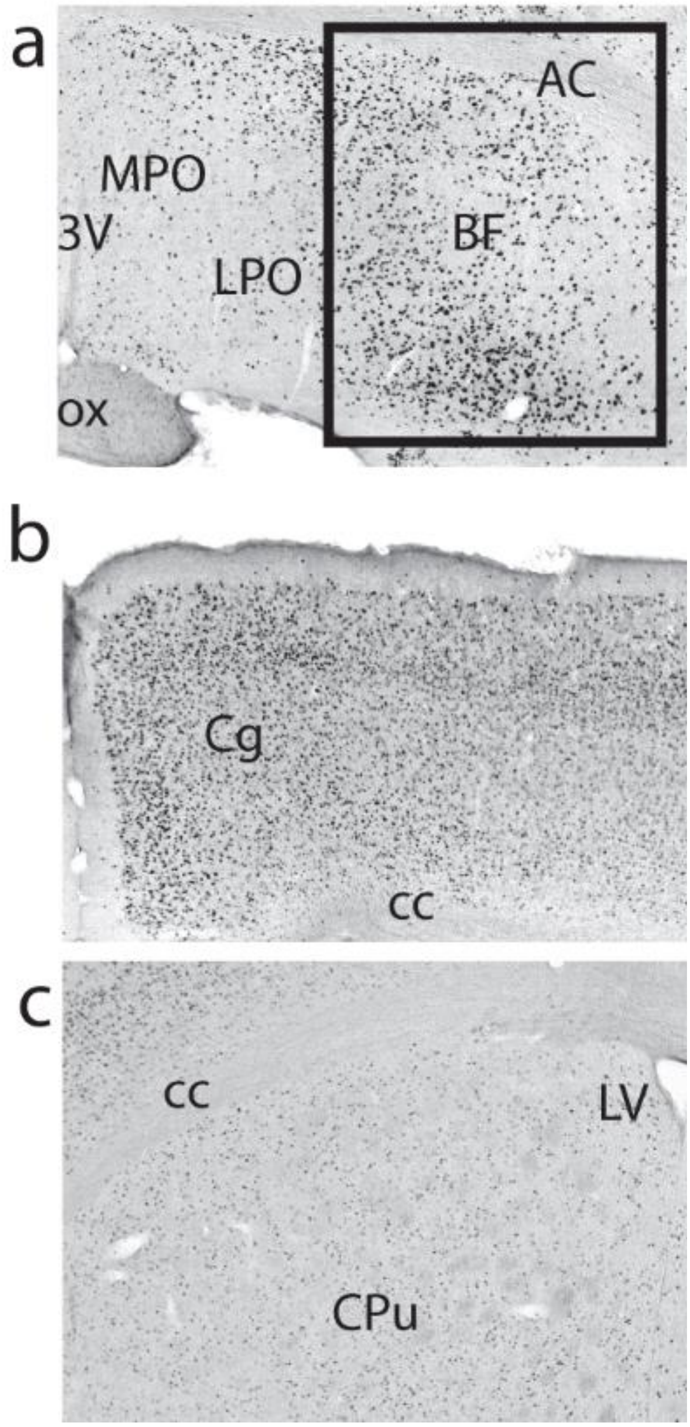

Supplementary Figure 4. c-Fos expression in the basal forebrain (BF) and neighboring preoptic region, cortex and striatum following CNO administration in Vgat-IRES-Cre mice expressing hM3Dq. CNO (0.3mg/kg, IP, ZT3 = 10 A.M.) induce robust c-Fos expression in BF neurons of Vgat-IRES-cre expressing hM3Dq. Of note, despite intense activation of BF neurons (black box), similarly robust c-Fos expression was not induced in neurons of the neighboring median or lateral preoptic areas (MPO, LPO), which instead showed only sparse c-Fos labeling (**a**). Consistent with the waking and fast cortical rhythms induced by CNO in these mice, however, robust c-Fos expression was observed in the cortex, in particular within the cingulate cortex (Cg) (**b**). Similar to the c-Fos labeling withing the MPO and LPO, only sparse c-Fos was observed in the dorsally situated striatum (**c**). 3L = third ventricle; AC = anterior commissure; cc = corpus callosum; CPu = caudate putamen; LV = lateral ventricle; ox = optic chiasma.

Supplementary Figure 5

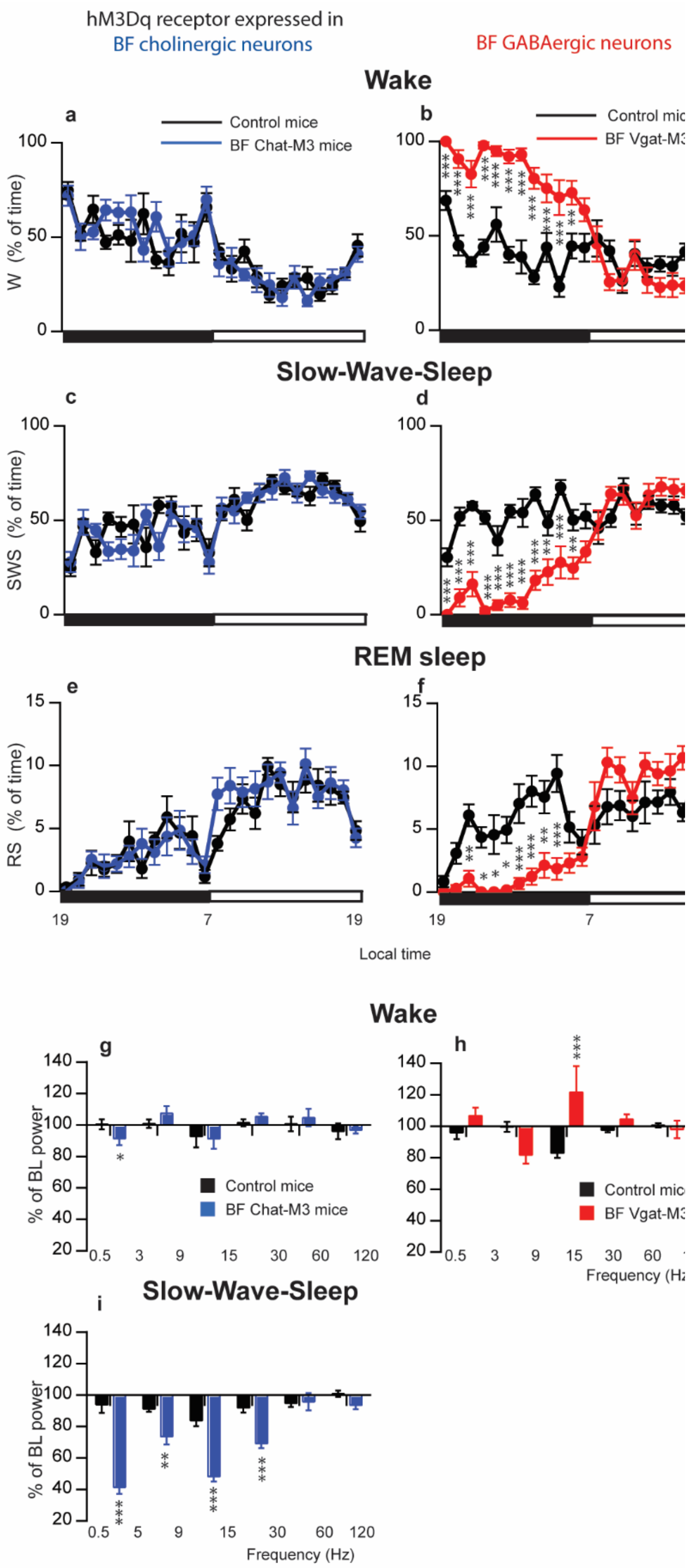

Supplementary Figure 5. The behavioral and EEG effects of ligand-driven activation of basal forebrain (BF) cholinergic or GABAergic neurons effect did not differ by injection time. (**a-f**) Hourly sleep-wake amounts following control or CNO (0.3 mg/kg, IP, ZT12 = 7 P.M.) injections in mice expressing hM3Dq receptors in BF cholinergic (n = 8) or GABAergic (n= 9) neurons. (**g-i**) Power density for the δ (0.5-3 or 0.5-5 Hz), θ (3-9 or 5-9 Hz), α (9-15 Hz), β (15-30 Hz), low γ (30-60 Hz) and high γ (60-120 Hz) frequency bands (± SEM) following control or CNO (0.3 mg/kg, IP, ZT12 = 7 P.M.) injections in mice expressing hM3Dq receptors in BF cholinergic (**g,i**; n = 8) or GABAergic (**h**; n= 8) neurons. Consistent with the results obtained with the day time injections, sleep-wake amounts were not affected by the CNO injection in in mice expressing hM3Dq receptors in BF cholinergic neurons but wake amount was largely and significantly increased in mice expressing hM3Dq receptors in BF GABAergic neurons. Moreover, delta to beta frequency bands were significantly decreased during SWS by CNO injection in mice expressing hM3Dq receptors in BF cholinergic neurons. A two-way ANOVA using the between-subjects factor of injection (control and CNO injection) and the within-subjects factors of time of day (hourly) or frequency band was used to analyze the percentage(s) of time spent in W, SWS and REM sleep or the EEG power in W and SWS, * $p < 0.05$, ** $p < 0.01$ and *** $p < 0.001$.

Supplementary Figure 6

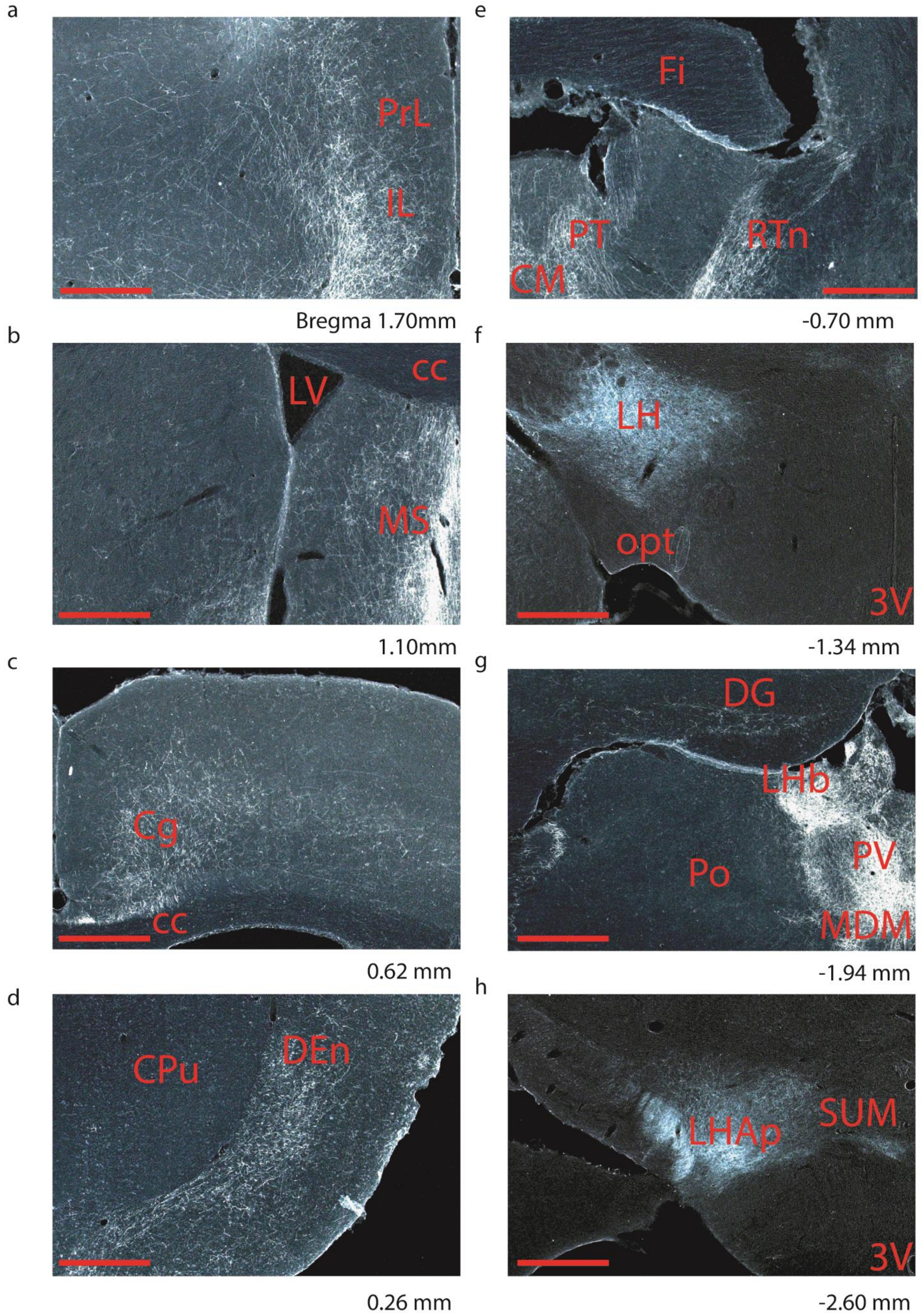

Supplementary Figure 6. Cre-dependent anterograde tracing to map axonal projections of basal forebrain (BF) GABAergic neurons. Injections of FLEX-ChR2-YFP were placed into the BF of Vgat-ires-Cre mice. After 4 post-surgical weeks, brains from these mice were processed using immunohistochemical methods to reveal the extent and location of BF GABAergic axonal projections. As shown in the figure panels, which run rostral to caudal, BF GABAergic neurons project to several cortical (**a, c, d**) and subcortical (**b, e, f, g, h**) targets, the latter including midline and intralaminar thalamic nuclei (**e, g**), reticular thalamus (**e**), lateral and medial habenula (**g**), lateral and posterior lateral hypothalamus and the supramammilarly region (**f, h**). Other postsynaptic targets not shown include the entorhinal cortex and ventral tegmental area. Scale bars = 500um. 3V = third ventricle; cc = corpus callosum; CM = centromedial thalamic nucleus; CPu = caudate putatmen (striatum); DEn = dorsal endopiriform; DG = dentate gyrus; Fi = fimbria of hippocampus; IL = infralimbic cortex; LH = lateral hypothalamus; LHAp = posterior lateral hypothalamic area; LHb = lateral habenular nucleus; LV = lateral ventricle; MDM = mediodorsal thalamic nucleus; MS = medial septum; Opt = optic tract; PrL = prelimbic cortex; PT = paratenial thalamus; Po = posterior thalamic nucleus; PV = paraventricular thalamus; RTn = reticular thalamus; SUM = supramammilary hypothalamus.

Supplementary Figure 7

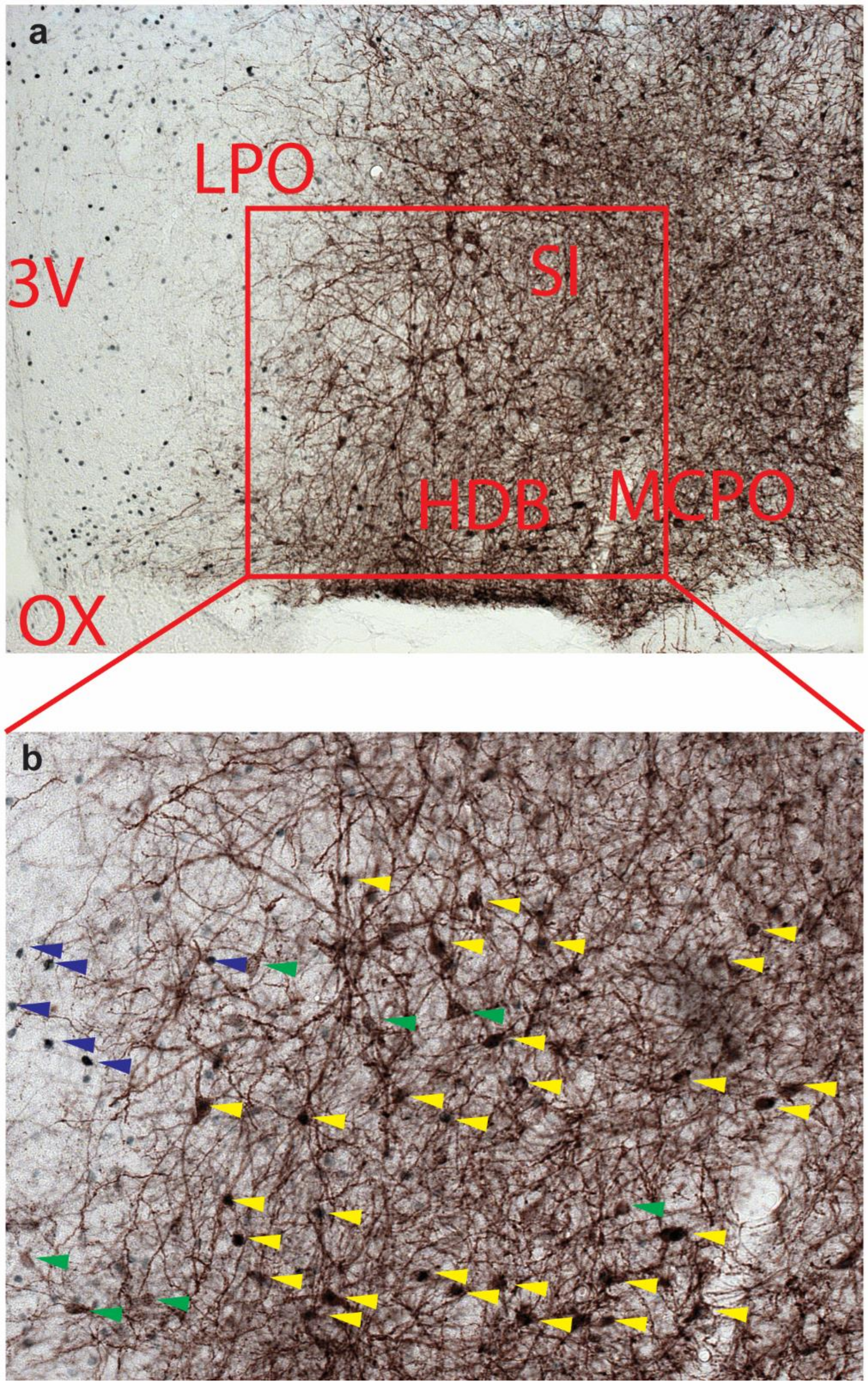

Supplementary Figure 7. Robust c-Fos activation in hM3Dq+ BF neurons following CNO injections in Vglut2-IRES-Cre mice. (**a**) coronal section from a hM3Dq-AAV injected Vglut2-IRES Cre mouse showing dense soma and neuropil labeling in the BF (mCherry+, brown) and nuclear c-Fos (black). (**b**) a higher power image of the region in the red box in (**a**) showing neurons expressing c-Fos alone (blue arrows), mCherry alone (green arrows) or both c-Fos and mCherry (yellow arrows). The large number of double-labeled neurons is consistent with robust CNO-induced activation (0.3mg/kg, IP, ZT3 = 10 A.M.) of hM3Dq+ neurons in the BF of Vglut2-IRES-Cre mice. 3V = third ventricle; AC = anterior commissure; ox = optic chiasma; LPO = lateral preoptic area; HDB = horizontal limb of the diagonal band; MCPO = magnocellular preoptic nucleus; SI = substantia innominata.

Supplementary Tables

Supplementary Table 1			Wake		SWS		REM sleep	
			**Control**	**hM3Dq**	**Control**	**hM3Dq**	**Control**	**hM3Dq**
Mice expressing hM3Dq in BF cholinergic neurons	**Light**	% of time	32.2±1.1	31.2±1.1	60.6±0.9	61.5±1.0	7.2±0.3	7.3±0.2
		Number	110.0±8.2	125.5±10.6	111.4±8.8	126.8±10.6	43.9±2.7	45.4±1.9
		Duration (min)	2.0±0.2	1.8±0.1	4.0±0.3	3.7±0.2	1.2±0.0	1.2±0.0
	**Dark**	% of time	57.4±2.5	54.5±2.6	39.6±2.3	42.1±2.3	3.0±0.3	3.3±0.4
		Number	71.3±6.8	90.8±7.3	70.7±6.7	90.0±7.3	23.0±2.2	23.7±2.6
		Duration (min)	6.9±0.8	5.1±0.6	3.9±0.3	3.5±0.2	0.9±0.1	1.0±0.1
Mice expressing hM3Dq in BF GABAergic neurons	**Light**	% of time	36.7±2.2	34.4±1.0	56.8±2.1	59.0±1.0	6.5±0.4	6.6±0.3
		Number	117.1±9.4	106.8±6.0	119.3±9.7	108.8±6.2	46.3±2.6	49.2±2.7
		Duration (min)	2.3±0.2	2.4±0.2	3.6±0.3	4.0±0.2	1.0±0.1	1.0±0.0
	**Dark**	% of time	53.4±2.8	52.1±2.4	42.4±2.4	43.7±2.0	4.2±0.5	4.2±0.4
		Number	87.1±7.8	76.6±4.3	87.3±7.9	76.6±4.4	30.0±3.4	28.1±3.3
		Duration (min)	4.9±0.6	5.1±0.4	3.6±0.2	4.2±0.2	1.0±0.1	1.1±0.0
Mice expressing hM3Dq in BF glutamatergic neurons	**Light**	% of time	34.2±1.8	37.1±1.9	59.0±1.6	56.1±1.8	6.8±0.4	6.8±0.5
		Number	111.6±5.6	90.7±4.6	112.8±6.1	91.9±4.5	43.4±3.5	45.3±3.4
		Duration (min)	2.1±0.2	3.0±0.3	3.8±0.2	4.5±0.2	1.1±0.1	1.1±0.0
	**Dark**	% of time	53.9±1.9	48.5±1.7	42.5±1.6	46.5±1.7	3.7±0.4	5.1±0.2
		Number	80.1±6.8	71.9±2.8	79.9±7.0	72.0±2.7	24.2±3.0	32.6±1.8
		Duration (min)	5.6±0.5	5.2±0.3	4.0±0.3	4.7±0.2	1.1±0.0	1.1±0.0
Mice expressing hM3Dq in thalamus glutamatergic neurons	**Light**	% of time	33.8±1.8	32.8±2.3	57.7±1.5	58.5±2.4	8.6±0.7	8.7±0.5
		Number	103.6±6.3	109.1±6.3	105.4±6.8	110.5±6.7	50.6±3.7	53.4±3.8
		Duration (min)	2.3±0.3	2.2±0.2	4.0±0.2	3.9±0.3	1.2±0.1	1.2±0.0
	**Dark**	% of time	56.7±1.3	52.9±3.0	39.0±1.3	42.1±2.8	4.3±0.3	5.0±0.6
		Number	66.6±5.2	84.1±5.9	66.3±5.1	83.9±5.9	27.9±2.4	30.9±3.3
		Duration (min)	6.7±0.7	4.7±0.4	4.5±0.4	3.7±0.3	1.1±0.0	1.2±0.1

Supplementary Table 1. In the absence of CNO, hM3Dq receptor expression in basal forebrain (BF) or thalamocortical (TC) neurons was without effect on the sleep-wake cycle. Percentage, number of bouts and mean (± SEM) bout duration (min) of spontaneous sleep-wake state (Wake, SWS and REM sleep) in mice expressing the hM3Dq receptor in BF cholinergic (ChAT, n = 14), GABAergic (Vgat, n = 13) and glutamatergic (Vglut2, n = 11) as well as in thalamus glutamatergic (n = 8) neurons as compared with non-cre-expressing littermate mice (n = 7, 8, 9 and 8, respectively). Averages are given for the 12-hour light period and the 12-hour dark period. A two-way ANOVA using the between-subjects factor of hM3Dq expression (control and BF ChAT-, Vgat- or Vglut2-hM3Dq expressing mice) and the within-subjects factors of time of day (light and dark periods) was used to analyze the percentage(s) of time spent in W, SWS and REM sleep, as well as the frequency and average bout durations of each stage during the light and dark periods. No significant differences were found between hM3Dq-expressing mice and non-cre-expressing littermates.

Supplementary Table 2			Wake		SWS		REM sleep	
			Vehicle	CNO	Vehicle	CNO	Vehicle	CNO
**Chat-IRES-cre**	**Time**	Latency (min)			22.7±1.3	22.0±3.9	58.8±6.1	72.3±14.1
	**10-19**	% of time	31.2±1.3	31.2±1.6	61.2±0.9	62.0±1.4	7.6±0.4	6.8±0.3
		Number	99.4±8.3	88.7±6.0	99.3±8.3	89.3±5.9	34.9±2.7	27.7±1.8
		Duration (min)	1.7±0.2	1.9±0.2	3.5±0.3	3.8±0.2	1.2±0.0	1.3±0.1
	**19-7**	% of time	56.4±2.3	56.4±2.4	40.0±1.9	39.9±2.0	3.5±0.4	3.6±0.4
		Number	85.1±9.3	83.4±8.9	85.4±9.5	83.1±9.0	27.7±2.5	26.1±2.0
		Duration (min)	5.4±0.7	5.6±0.9	3.6±0.3	3.7±0.4	0.9±0.1	1.0±0.1
**Vgat-IRES-cre**	**Time**	Latency (min)			27.2±3.3	25.5±5.4	58.1±5.3	49.4±11.2
	**10-19**	% of time	36.1±1.1	35.5±1.9	57.4±1.3	57.9±1.7	6.5±0.6	6.6±0.5
		Number	68.9±12.3	71.0±9.0	88.1±4.7	84.9±3.1	56.6±8.9	48.1±9.6
		Duration (min)	1.6±0.2	2.2±0.4	3.6±0.2	3.5±0.3	1.5±0.3	1.3±0.2
	**19-7**	% of time	50.5±2.4	49.3±1.9	45.4±2.2	46.1±1.7	4.1±0.4	4.6±0.4
		Number	94.1±7.8	93.1±3.8	94.4±8.1	93.3±3.9	29.0±3.2	30.4±2.3
		Duration (min)	4.3±0.5	3.9±0.2	3.5±0.2	3.6±0.3	1.0±0.0	1.1±0.0
**Vglut2-IRES-cre**	**Time**	Latency (min)			45.4±10.8	21.5±2.9	77.4±14.1	53.5±9.8
	**10-19**	% of time	38.1±1.7	34.7±1.1	54.0±5.5	57.1±0.6	7.9±0.9	8.2±0.7
		Number	80.3±6.2	84.7±2.8	80.0±6.2	84.4±2.7	34.6±2.9	36.6±3.8
		Duration (min)	2.7±0.2	2.2±0.1	3.7±0.2	3.7±0.1	1.2±0.1	1.2±0.1
	**19-7**	% of time	48.7±1.2	48.3±1.8	45.8±0.8	46.4±2.0	5.6±0.6	5.3±0.3
		Number	87.6±4.1	89.6±5.2	87.4±4.2	89.3±5.2	36.3±3.0	34.6±2.6
		Duration (min)	4.2±0.2	4.2±0.4	3.8±0.2	3.7±0.2	1.1±0.1	1.1±0.0

Supplementary Table 2. In the absence of hM3Dq receptor expression in basal forebrain (BF) or thalamocortical (TC) neurons, CNO was without effect on the sleep-wake cycle. Sleep latencies, percentage, number of bouts and mean (± SEM) bout duration (min) of sleep-wake state (Wake, SWS and REM sleep) in Chat-IRES-cre (n = 7), Vgat-IRES-cre (n = 7) or Vglut2-IRES-cre (n = 7) mice following CNO (0.3 mg/kg, IP, ZT3) or vehicle injections. Averages are given for the 9-hour light period following the injection (10-19) and the 12-hour of the dark period (19-7) A two-way ANOVA using the between-subjects factor of injection (control and CNO injection) and the within-subjects factors of time of day (10-19 and 19-1) was used to analyze the percentage(s) of time spent in W, SWS and REM sleep, as well as the frequency and average bout durations of each stage during the different periods. Paired t tests were used to analyze latency to SWS or REM sleep. No significant differences were observed, confirming that CNO is pharmacologically inert in the absence of its receptor.

Supplementary Table 3

	mCherry+		
	Chat-IRES-cre	Vglut2-IRES-cre	Vgat-IRES-cre
**Cell marker**	92 ± 2	83 ± 4	75 ± 3
**c-fos (%)**	92 ± 5	88 ± 3	85 ±2
**mCherry+ (%) non-cre-expressing mice**	ND	ND	ND

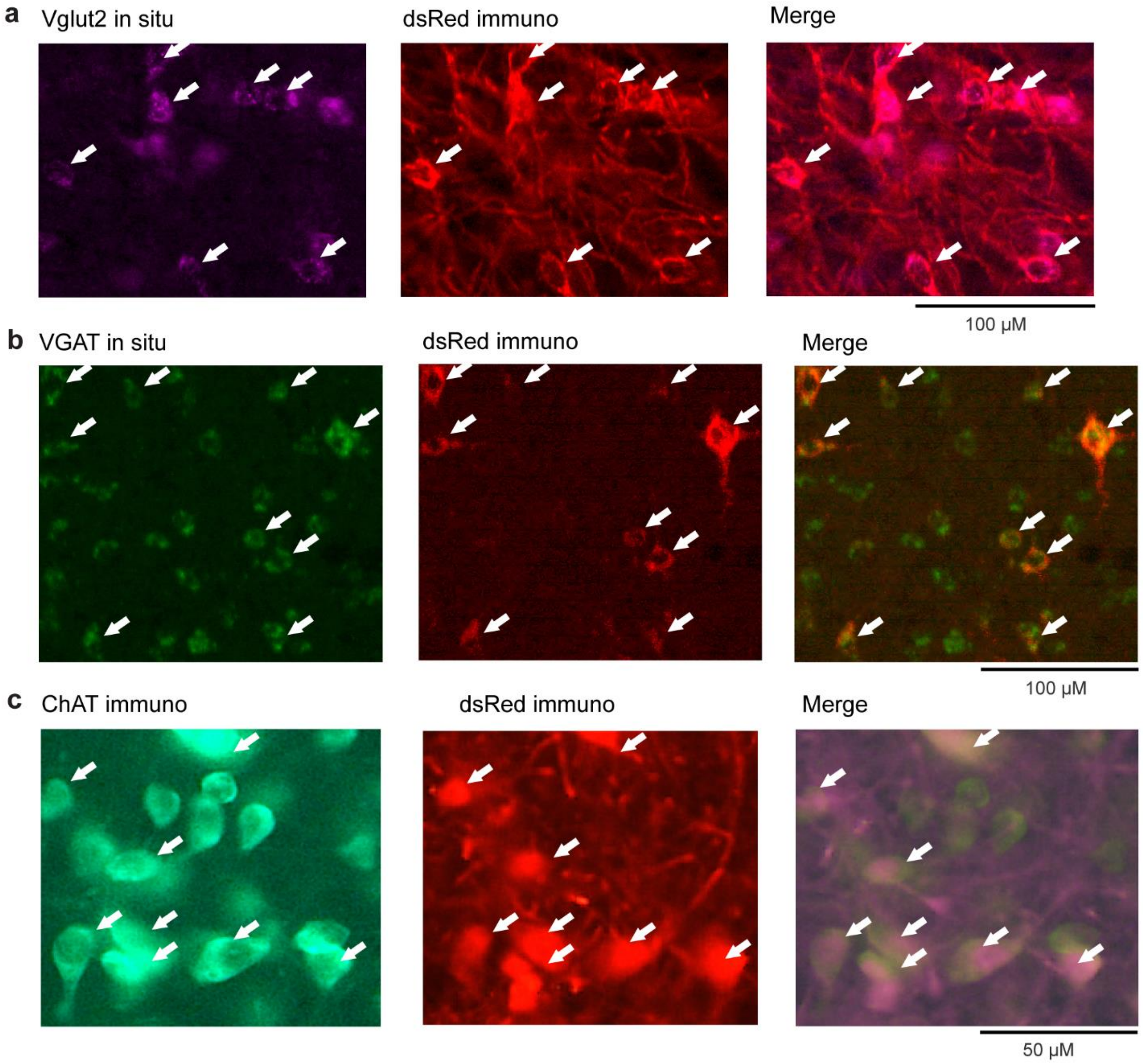

Supplementary Table 3. AAV validation and quantification of c-Fos colocalization.
The extent of colocalization of hM3Dq (mCherry+ neurons) following AAV-mediated delivery of hSyn-DIO-hM3D-mCherry to the basal forebrain (BF), and Chat, Vgat, and Vglut2, respectively, was quantified. Cell counting was performed by placing a grid (approximating the red box in Supplementary Figure 1b) over the individual sections (n=5 sections/brain, spanning ~ Bregma +0.26 to -0.25) and counting the number of mCherry+ (red), ChAT+, Vgat+ or Vglut2+ (green) cells and then dividing by the total number of green cells, which yielded the percentage of cells that co-localized mCherry (fusion protein with hM3Dq and hence proxy for detection) for the respective cell subtypes. Our analysis revealed excellent transfection of the AAV in the BF of all mouse lines. Importantly, dual-labeling was observed in nearly all hM3Dq+ neurons in all 3 cre-driver mouse lines, with only occasional single-labeled hM3Dq+ cells observed. Hence we cannot exclude the possibility that very minor ectopic expression of hM3Dq occurred in the BF of the injected mouse lines, in particular as we did not perform confocal-based analysis, i.e., expression or co-localization beyond on limits of detection. In contrast, hM3Dq+ cells were not detected in non-cre-expressing littermate mice with BF-directed injections of hM3Dq-AAV, for any of the mouse lines (not shown). CNO-induced c-Fos expression was quantified for all 3 mouse lines, using the same approach as described above. Our analysis revealed robust c-Fos activation, in all mouse lines, within hM3Dq+ cells by CNO [example for Vglut2-IRES-cre mice expressing hM3Dq in BF neurons is shown in Supplementary Figure 7]. Located above (below Table S3) are example images showing specificity of

labeling in the BF of (**a**) Vglut2, (**b**) Vgat and (**c**) ChAT cre mice following injections of *hSyn-DIO-hM3Dq-mCherry-AAV*. ND = not detected.

Supplementary Table 4

			Wake		SWS		REM sleep	
			**Vehicle**	**CNO**	**Vehicle**	**CNO**	**Vehicle**	**CNO**
**Mice expressing hM3Dq in BF cholinergic neurons**	**Time**	Latency (min)			21.9±4.0	29.3±3.7	59.0±8.3	89.9±13.4^{A}
	**10-19**	% of time	29.0±1.1	32.4±1.1	63.7±1.0	59.8±1.0	7.4±0.2	7.7±0.3
		Number	98.2±3.8	124.0±8.2^{B}	100.4±3.9	125.5±8.2^{B}	33.6±1.7	31.9±1.6
		Duration (min)	1.4±0.1	1.2±0.1	3.5±0.1	2.7±0.2^{A}	1.2±0.0	1.3±0.0
	**19-1**	% of time	56.6±3.2	53.8±3.2	40.7±3.0	43.4±3.0	2.7±0.4	2.8±0.3
		Number	44.2±2.9	58.5±5.0	43.8±2.9	58.6±5.2	10.5±1.0	11.1±1.0
		Duration (min)	5.1±0.6	3.5±0.3^{A}	3.4±0.2	3.0±0.3	0.9±0.1	1.0±0.1
	**1-7**	% of time	50.5±2.8	50.3±3.2	45.4±2.4	45.8±2.8	4.1±0.5	3.9±0.6
		Number	45.8±3.1	48.2±4.4	45.7±3.0	48.2±4.4	15.2±1.5	13.3±1.1
		Duration (min)	4.6±0.5	4.2±0.5	3.7±0.2	3.6±0.5	1.0±0.1	1.0±0.1
**Mice expressing hM3Dq in BF glutamatergic neurons**		Latency (min)			31.0±3.8	27.7±3.3	78.9±6.4	91.3±10.0
	**10-19**	% of time	33.1±1.2	33.2±1.7	59.6±1.0	60.0±1.6	7.3±0.4	6.9±0.5
		Number	86.7±4.8	82.0±4.4	87.9±4.9	83.2±4.4	36.3±1.8	29.6±2.4^{A}
		Duration (min)	1.8±0.1	1.7±0.2	3.8±0.2	4.0±0.2	1.1±0.0	1.3±0.0
	**19-1**	% of time	52.7±2.2	44.4±1.7^{A}	43.5±2.0	50.2±1.5^{A}	3.8±0.4	5.4±0.4^{A}
		Number	39.5±3.5	43.5±1.8	39.8±3.4	43.5±1.7	14.4±1.3	16.8±1.4
		Duration (min)	5.3±0.5	4.0±0.2^{A}	4.2±0.3	4.1±0.2	1.0±0.1	1.1±0.1
	**1-7**	% of time	36.8±1.7	42.3±2.8	56.4±1.6	51.8±2.5	6.8±0.5	5.9±0.4
		Number	45.4±3.1	42.5±2.2	45.2±3.2	42.5±2.4	22.3±1.6	21.7±1.7
		Duration (min)	3.0±0.3	3.9±0.5	4.7±0.3	4.5±0.1	1.1±0.0	1.0±0.1
**Mice expressing hM3Dq in BF GABAergic neurons**		Latency (min)			28.6±3.3	238.5±36.0^{C}	68.1±7.3	317.7±28.7^{C}
	**7-10 Before CNO injection**	% of time	33.8±3.3	38.2±2.8	59.7±3.1	56.1±2.6	6.5±0.4	5.7±0.4
		Number	27.9±2.7	26.8±1.9	28.2±2.7	26.8±1.9	13.5±1.2	10.8±1.2
		Duration (min)	2.6±0.5	2.6±0.3	4.1±0.3	4.2±0.4	0.9±0.1	1.0±0.1
	**10-19**	% of time	34.0±1.2	76.2±3.5^{C}	59.3±1.1	22.0±3.4^{C}	6.7±0.3	1.8±0.3^{C}
		Number	84.8±4.2	43.8±6.7^{C}	85.3±4.3	43.6±6.7^{C}	39.0±2.4	10.3±1.8^{C}
		Duration (min)	2.3±0.2	16.0±4.5	3.8±0.3	2.9±0.3^{A}	1.0±0.1	1.1±0.1
	**19-1**	% of time	53.5±2.5	75.9±5.1^{C}	42.8±2.1	22.0±4.6^{C}	3.7±0.4	2.1±0.6^{A}
		Number	41.6±2.7	29.9±5.2	41.3±2.8	29.5±5.3	12.6±1.5	7.0±2.1
		Duration (min)	5.4±0.5	52.2±33.1^{A}	3.9±0.2	2.3±0.3^{C}	1.1±0.1	0.7±0.2^{B}
	**1-7**	% of time	40.6±2.8	44.6±5.2	53.6±2.5	48.4±4.4	5.8±0.4	6.9±0.9
		Number	44.4±2.5	53.8±4.5	44.7±2.5	54.6±4.5	20.2±1.8	23.0±3.2
		Duration (min)	3.0±0.3	3.1±0.7	4.6±0.3	3.4±0.3^{B}	1.1±0.0	1.1±0.1
	**D+1 7-19**	% of time	33.6±1.3	26.9±1.1	59.8±1.2	65.0±1.0	6.6±0.3	8.1±0.5
		Number	103.8±6.1	129.7±6.0^{C}	106.1±6.4	131.0±6.2^{C}	48.4±2.7	67.9±5.3^{C}
		Duration (min)	2.5±0.2	1.7±0.2	4.1±0.2	3.5±0.3	1.0±0.0	0.9±0.0
**Mice expressing hM3Dq in glutamatergic Thalamus neurons**		Latency (min)			25.4±4.0	42.1±14.9	56.6±7.2	86.3±17.2
	**10-19**	% of time	34.0±2.1	37.0±3.8	57.6±2.1	54.3±4.2	8.4±0.5	8.7±0.8
		Number	114.1±9.0	118.1±11.5	169.6±27.2	157.5±22.3	59.9±6.4	57.5±6.4
		Duration (min)	1.7±0.1	1.6±0.1	2.2±0.4	2.3±0.4	0.8±0.1	0.9±0.1
	**19-1**	% of time	56.9±3.3	55.8±3.5	39.3±3.2	39.2±3.6	3.8±0.9	5.0±0.7
		Number	45.3±4.9	51.3±4.2	44.9±4.8	50.8±4.1	12.1±2.9	16.9±2.9
		Duration (min)	5.1±1.0	4.4±0.6	3.3±0.3	2.8±0.2	1.1±0.1	1.1±0.1
	**1-7**	% of time	48.4±3.6	43.3±3.1	45.9±3.4	48.3±2.9	5.7±0.6	8.5±0.5^{B}
		Number	44.3±2.7	48.6±3.2	44.3±2.8	49.1±3.2	17.4±1.9	26.4±2.4^{C}
		Duration (min)	4.1±0.4	3.1±0.2	3.9±0.3	3.7±0.4	1.3±0.1	1.2±0.1

Supplementary Table 4. Ligand-driven activation of basal forebrain (BF) cholinergic, glutamatergic or GABAergic neurons or thalamocortical (TC) glutamatergic neurons differentially affects the sleep-wake cycle. Sleep latencies, percentage, number of bouts and mean (± SEM) bout duration (min) of sleep-wake state (Wake, SWS and REM sleep) in mice expressing hM3Dq receptor in cholinergic (n = 14), GABAergic (n = 13) or glutamatergic (n = 11) neurons of the BF as well as mice expressing hM3Dq receptor in glutamatergic (n = 8) neurons of the thalamus, following CNO (0.3 mg/kg, IP, ZT3) or vehicle injections. Averages are given for the 9-hour light period following the injection (10-19), the 6-hour of the first half of the dark period (19-1) and the 6-hour of the second half of the dark period (1-7). Given the large and sustained increase of wake amount following CNO injection in mice expressing hM3Dq receptor in GABAergic neurons of the basal forebrain, the subsequent 12-hour light period (D+1 7-19) was also analyzed. A two-way ANOVA using the between-subjects factor of injection (control and CNO injection) and the within-subjects factors of time of day (10-19, 19-1, 1-7 and/or D+1 7-19) was used to analyze the percentage(s) of time spent in W, SWS and REM sleep, as well as the frequency and average bout durations of each stage during the different periods; paired T tests were used to analyze the latency to SWS or REM sleep. ^{A} $p < 0.05$, ^{B} $p < 0.01$ and ^{C} $p < 0.001$ as compared with control injection.

Supplementary Table 5

	Time		Wake		SWS		REM sleep	
			Vehicle	CNO	Vehicle	CNO	Vehicle	CNO
Mice expressing hM3Dq in BF cholinergic neurons	Time	Latency (min)			26.9±5.2	29.6±7.9	121.8±22.7	145.1±34.5
	19-7	% of time	53.3±3.0	56.6±3.2	43.9±2.8	40.7±2.9	2.8±0.4	2.6±0.4
		Number	106.0±12.9	115.1±13.8	105.8±12.8	114.3±13.8	20.5±3.8	18.0±2.0
		Duration (min)	3.9±0.5	4.1±0.6	3.3±0.3	2.8±0.3^{A}	1.0±0.1	1.1±0.1
	7-19	% of time	30.6±0.6	28.6±1.2	62.2±0.6	63.4±1.2	7.2±0.1	8.0±0.4
		Number	134.0±13.2	141.4±11.6	134.6±13.2	143.1±11.4	44.4±2.1	49.5±3.7
		Duration (min)	1.7±0.2	1.4±0.1	3.4±0.3	3.3±0.3	1.2±0.1	1.2±0.1
Mice expressing hM3Dq in BF GABAergic neurons		Latency (min)			26.7±6.7	307.0±77.8^{B}	70.8±9.9	467.0±90.3^{B}
	19-7	% of time	42.7±1.6	84.5±3.3^{C}	51.9±1.3	14.4±3.0^{C}	5.4±0.4	1.0±0.3^{C}
		Number	104.0±3.9	61.4±14.5^{C}	103.7±5.8	61.1±14.6^{C}	40.6±3.4	8.1±2.7^{C}
		Duration (min)	3.1±0.2	29.7±15.1^{A}	3.6±0.1	2.2±0.4^{C}	1.0±0.0	0.9±0.2
	7-19	% of time	36.4±1.4	28.3±0.9^{B}	56.7±1.2	62.4±1.0^{A}	6.9±0.5	9.3±0.5^{C}
		Number	134.4±7.0	130.2±6.8	135.4±6.8	131.9±7.0	57.2±5.5	71.9±4.6^{B}
		Duration (min)	1.9±0.1	1.6±0.1^{A}	3.1±0.2	3.5±0.2	0.9±0.0	0.9±0.0
	D+1 19-7	% of time	53.4±2.7	45.4±3.7^{B}	42.2±2.2	49.4±3.1^{A}	4.4±0.6	5.2±0.7
		Number	76.7±5.8	96.4±7.3	76.8±5.7	97.0±7.5	32.0±5.0	37.1±4.8
		Duration (min)	5.3±0.6	3.8±0.8	4.1±0.3	3.7±0.2	1.1±0.0	1.0±0.1
Mice expressing hM4Di in BF GABAergic neurons		Latency (min)			55.4±22.6	14.6±2.7	73.1±23.4	4.9±13.8
	19-22	% of time	69.3±7.2	48.7±4.8^{B}	28.6±6.8	47.6±4.3^{B}	2.0±0.5	3.8±0.7
		Number	12.3±2.5	20.6±2.3	11.7±2.5	20.3±2.1	4.7±1.0	8.6±1.8
		Duration (min)	34.9±25.0	4.6±0.8	3.7±0.6	4.0±0.1	0.8±0.1	0.7±0.1
	22-7	% of time	51.5±3.2	54.1±1.5	43.0±2.8	40.4±1.6	5.5±0.6	5.5±0.3
		Number	56.6±5.7	50.9±3.0	56.7±5.9	51.9±3.3	27.7±2.4	26.4±2.3
		Duration (min)	5.5±1.1	6.0±0.5	4.3±0.2	4.4±0.4	1.1±0.0	1.2±0.1
	7-19		35.8±3.0	40.7±3.2	56.1±2.9	51.4±3.1	8.1±0.4	7.9±0.5
			104.1±5.2	96.9±3.2	105.3±5.3	97.4±2.9	60.1±4.5	58.3±3.7
			2.5±0.3	3.2±0.3	3.8±0.2	3.8±0.3	1.0±0.0	1.0±0.0

Supplementary Table 5. Ligand-driven activation of basal forebrain (BF) cholinergic or GABAergic neurons expressing hM3Dq produced the same effect on sleep-wake at both ZT3 (10am) and ZT12 (7pm), whereas inhibition of BF GABAergic neurons expressing hM4Di produced a strong increase in NREM at ZT12. Sleep latencies, percentage, number of bouts and mean (± SEM) bout duration (min) of sleep-wake state (Wake, SWS and REM sleep) in mice expressing the hM3Dq receptor in BF cholinergic (n = 8) or GABAergic neurons (n = 9) as well as in mice expressing the hM4Di receptor in BF GABAergic neurons (n = 7), following CNO (0.3 mg/kg, IP, ZT12 = 7 P.M.) as compared with vehicle. Averages are given for the dark period (19-7), the light period (7-19), the following dark period (D+1 19-7), the 3 first hours of the dark period (19-22) followed by the rest of the dark period (22-7). A two-way ANOVA using the between-subjects factor of injection (control and CNO injection) and the within-subjects factors of time of day (light and dark periods) was used to analyze the percentage(s) of time spent in W, SWS and REM sleep, as well as the frequency and average bout durations of each stage during the light and dark periods; paired t tests were used to analyze the latency to SWS and REM sleep. ^{A} $p < 0.05$, ^{B} $p < 0.01$ and ^{C} $p < 0.001$ as compared with control injection.
